# Puf-A promotes cancer progression by interacting with nucleophosmin in nucleolus

**DOI:** 10.1038/s41388-021-02138-0

**Published:** 2022-01-09

**Authors:** Huan-Chieh Cho, Yenlin Huang, Jung-Tung Hung, Tsai-Hsien Hung, Kai-Chun Cheng, Yun-Hen Liu, Ming-Wei Kuo, Sheng-Hung Wang, Alice L. Yu, John Yu

**Affiliations:** 1 Institute of Stem Cell and Translational Cancer Research, Chang Gung Memorial Hospital at Linkou, Taoyuan, Taiwan; 2 Department of Anatomic Pathology, Chang Gung Memorial Hospital at Linkou, Taoyuan, Taiwan; 3 Department of Surgery, Chang Gung Memorial Hospital at Linkou, Taoyuan, Taiwan; 4 Genomics Research Center, Academia Sinica, Taipei, Taiwan; 5 Department of Pediatrics, University of California San Diego Medical Center, San Diego, CA USA; 6 Institute of Cellular and Organismic Biology, Academia Sinica, Taipei, Taiwan

**Keywords:** Non-small-cell lung cancer, Prognostic markers

## Abstract

Previously, we identified Puf-A as a novel member of Puf-family RNA-binding proteins; however, its biological functions remain obscure. Analysis of tumor samples of non-small cell lung cancer (NSCLC) showed that high Puf-A expression correlated with high histology grade and abnormal p53 status. Kaplan–Meier curve for overall survival revealed high expression of Puf-A to predict poor prognosis in stage I NSCLC. Among patients with colorectal cancer, high Puf-A expression also showed an adverse impact on overall survival. In lung cancer cell lines, downregulation of p53 increased Puf-A expression, and upregulation of p53 dampened its expression. However, luciferase reporter assays indicated that *PUF-A* locus harbored the p53-response element, but regulated Puf-A transcription indirectly. In vivo suppression of p53 in CCSP-rtTA/TetO-Cre/LSL-Kras^G12D^/p53^flox/flox^ conditional mutant mice accelerated the progression of the Kras^G12D^-driven lung cancer, along with enhanced expression of Puf-A. Importantly, intranasal delivery of shPuf-A to the inducible Kras^G12D^/p53^flox/flox^ mice suppressed tumor progression. Puf-A silencing led to marked decreases in the 80S ribosomes, along with decrease in S6 and L5 in the cytoplasm and accumulation in the nucleolus. Based on immunofluorescence staining and immunoprecipitation studies, Puf-A interacted with NPM1 in nucleolus. Puf-A silencing resulted in NPM1 translocation from nucleolus to nucleoplasm and this disruption of NPM1 localization was reversed by a rescue experiment. Mechanistically, Puf-A silencing altered NPM1 localization, leading to the retention of ribosomal proteins in nucleolus and diminished ribosome biogenesis, followed by cell-cycle arrest/cell death. Puf-A is a potential theranostic target for cancer therapy and an important player in cancer progression.

## Introduction

Through comparative evolutionary genomic analysis, we previously identified a novel gene, Puf-A (also known as KIAA0020 or PUM3). It belongs to the PUF family and highly expresses in primordial germ cells [1]. Human Pumilio 1 (PUM1), a classical PUF protein, recognizes specifically eight RNA bases by the side chains in its eight α-helical PUM repeats [2] which are highly conserved among classical PUF proteins. However, crystal structures of Puf-A revealed that it has 11 PUM repeats arranged in an L-like shape [3], suggesting Puf-A displays distinctive features. For example, fluorescence polarization assays showed that, unlike the classical PUF proteins, Puf-A bound with no apparent specificity to single- or double-stranded RNA or DNA [3]. On the other hand, Puf6, the yeast orthologue of the human Puf-A, is involved in ribosome biogenesis [4]. Even though Puf6 and Puf-A share only ~24% sequence identity, it is possible that human Puf-A may also play a role in ribosome biogenesis, but act differently. If so, Puf-A may contribute to tumorigenesis and tumor promotion, given the crucial importance of ribosome biogenesis for sustaining tumor cell growth. A single report showed higher expression of Puf-A in invasive breast cancer specimens than ductal carcinoma in situ [5], but it did not address its prognostic value nor possible involvement in ribosome biogenesis.

Many nucleolar factors, such as nucleophosmin (NPM1, also known as B23) and ribosomal proteins, participate in cancer growth in which robust ribosome biosynthesis is a hallmark [6]. NPM1, which is found in granular regions of nucleolus [7], plays a critical role in ribosome biogenesis by shuttling ribosomal proteins. Overexpression of NPM1 has been found in many cancers [8–10] where it induced c-Myc oncogenic action in the extranucleolar nucleoplasm [11] and influenced oxidative stress homeostasis by regulating peroxiredoxin 6 [12]. Puf-A was also found to possess amino acid residues in 106 to 118 similar to a putative nucleolar localization signal [13]. Whether Puf-A interacts with NPM1 in the nucleolus to affect the proliferation of cancer cells is unclear.

Ribosomal protein-MDM2-p53 signaling pathways have been found to be involved in p53 stability. *TP53* encoding the p53 protein is the most frequently altered gene in human tumors [14]. p53, a nuclear transcription factor, is expressed at very low level under normal conditions, due to proteasomal degradation by E3 ubiquitin protein ligase MDM2 [15, 16]. Upon DNA damage, p53 is phosphorylated and dissociated from MDM2/p53 complex, leading to regulation of many genes that are involved in cell-cycle arrest and apoptosis [17, 18]. The interaction between p53 and MDM2 can be disrupted by NPM1 when NPM1 translocates from the nucleolus to nucleoplasm where NPM1 bound to MDM2 to induce p53 accumulation [19]. However, mechanisms underlying NPM1 translocation are still unclear.

Here, we examined Puf-A expression and its correlation with clinical outcome in lung and colorectal cancers, investigated its transcriptional regulation, and elucidated its role in cancer proliferation, at least in part via interaction with NPM1. Our findings offer valuable insights into the potential theranostics value of Puf-A for cancer prognosis and therapy.

## Results

### High expression of Puf-A correlates with shortened survival in cancer patients

**Fig. 1:**
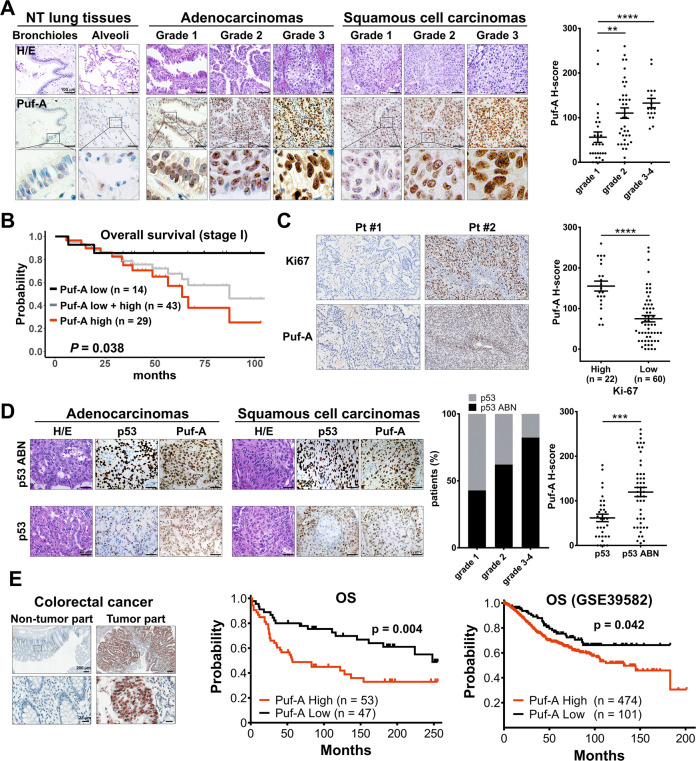
Clinical relevance of Puf-A expression in human cancers. **A** Tissue sections from patients with different grades of lung cancer and non-tumor (NT) region were used for hematoxylin and eosin staining and IHC staining with monoclonal antibody against Puf-A. The enlarged images of Puf-A staining are shown in the bottom panels. Puf-A expression level of each sample was presented using a histological scoring system, H-score (right panel). Each dot represents one sample and horizontal black lines represents mean ± SEM. **p < 0.01 and ***p < 0.001 by one-way ANOVA with Dunn’s multiple comparisons test. Scale bars: 100 µm. **B** The Kaplan–Meier analysis of overall survival of patients with stage I lung cancer (gray line) and of patients with the high (red line) and low (black line) expression of Puf-A. **C** Increased Ki-67 expression in lung ADC with high expression of Puf-A. Representative immunostainings for Ki-67 of patients with low (pt #1) and high (Pt #2) Puf-A expression were shown. Right panel is individual Puf-A H-score and mean ± SEM levels of both Ki-67 high (*n* = 22) and low (*n* = 60) in 82 lung cancer specimens. Scale bars: 200 µm. ****p < 0.0001 (two-tailed Mann–Whitney test). **D** Immunohistochemical analysis of p53 expression in tissue sections of NSCLC including ADC and SCC. The intensity of p53 staining was divided into two groups: “p53 abnormal (ABN)” refers to tumor cells with negative or strong nuclear p53 staining, and “p53” refers to those with weak staining. The percentages of p53 and p53 ABN in patients with different grades of lung cancer is shown in the middle panels. Right panel is individual Puf-A H-score and mean ± SEM levels of both p53 (*n* = 33) and p53 ABN (*n* = 49) in 82 lung cancer specimens. Scale bars: 200 µm. ***p < 0.001 (two-tailed *t* test). **E** Tissue sections from patients with colorectal cancers were stained with monoclonal antibody against Puf-A for immunohistochemical analysis. The expression of Puf-A in tumor and non-tumor parts was shown. The enlarged images of Puf-A staining are shown in the bottom panel. Middle panel is Kaplan–Meier curves for overall survival (OS) in colorectal cancer patients with high (H-score ≧25) and low (H-score <25) Puf-A expression. In the right panel, a RNA microarray database, GSE39582, was used to analyze the prognostic performance of Puf-A. *P* value was calculated using log-rank test.

We examined the expression of Puf-A protein in 82 NSCLC tumors, including 64 adenocarcinomas (ADCs), 14 squamous cell carcinomas (SCCs), 3 large cell carcinomas, and 1 sarcomatoid carcinoma, by immunohistochemistry (IHC) (Fig. 1A). Puf-A was readily detectable in both ADCs and SCCs, but barely discernible in adjacent non-tumor lung tissues. Low grade tumors showed conspicuous expression of Puf-A in the nucleoli, while grade 3 tumor cells exhibited Puf-A expression in nucleoli and nucleoplasm (Fig. 1A). The Puf-A expression was presented as H-score and correlated with clinical characteristics of the patients (Table S1). We found that Puf-A expression rose significantly with increasing histology grade in H-score (*p* = 0.0017 for grade 1 vs. grade 2; *p* < 0.0001 for grade 1 vs. grade 3–4). These findings are also in line with analysis of the datasets, GSE68571, which showed that *PUF-A* RNA was significantly higher in grade 2 and grade 3 tumors than in grade 1 tumor (Fig. S1).

Kaplan–Meier curve for overall survival (OS) revealed that Puf-A expression was highly prognostic (Fig. 1B) for patients with stage I NSCLC (Table S2). With median follow up of 2.5 years (0.5–7.2 years), the 5-year OS rate of stage 1 NSCLC patients was 67.8% (95% CI, 53.6–85.6%; gray line). This 5-year OS rate was significantly lower for those patients with high Puf-A expression, 57.0% (95% CI 38.5–84.3%, red line) compared to those with low Puf-A expression (85.7%, 95% CI 69.2–100%, black line, *p* = 0.038). We further stratified the stage 1 NSCLC into p53 normal and p53 mutant/silenced. As shown in Fig. S2, OS was best for patients with low Puf-A and normal p53 and worst for patients with high Puf-A and abnormal p53 (*p* = 0.09). However, the sample size was quite small for each stratified group. Therefore, we used stages 1 NSCLC from Pan-Cancer Atlas of TCGA for similar analysis. We found that patients with high *PUM3* (Puf-A) have inferior PFS (*p* = 0.0027) and OS (*p* = 0.05) as compared to those with low *PUM3* as shown in Fig. S3A, B. When stratified into four groups according to their *TP53* status and *PUM3* level, patients with *PUM3* high and *TP53* mutation had the worst PFS and OS, and those with *PUM3* low and *TP53* WT had the best outcome (Fig. S3C, D, PFS *p* < 0.001; OS *p* = 0.003). As to the GSE39582 database of CRC, p53 status was available in only 344 samples. Similar findings were observed with worst outcome for patients with *PUM3* high and *TP53* mutation (Fig. S4). These findings further strengthen the impact of Puf-A on cancer progression.

Next, to examine whether Puf-A expression was associated with the proliferative activity of lung cancer cells, we assessed the proliferating lung cancer by IHC with an antibody against Ki-67. With a total of 82 samples, H-score showed significantly higher Puf-A expression in Ki-67^high^ specimens (155.2 ± 12.3) than in Ki-67^low^ samples (74.8 ± 7.8, *p* < 0.0001) (Fig. 1C).

It was reported that TP53 gene abnormality occurred in 51.8% and 79.3% of lung ADC and SCC, respectively, and contributed to poor clinical outcome [14]. We determined the expression of p53 protein in tumor specimens by IHC (*n* = 82, Table S1) to explore the possible interplay between p53 and Puf-A. Since normal p53 had a very short half-life in cells, we considered weak staining of p53 protein as normal p53 expression; in contrast, strong or no staining of p53 as abnormal p53 expression in IHC analysis [20]. As shown in Fig. 1D, lung ADC with strong p53 staining (abnormal p53) often showed strong Puf-A staining, and those with weak p53 staining (normal p53) displayed weak Puf-A staining. Similar results were found for SCC. Moreover, the incidence of abnormal p53 expression in NSCLC tumor increased significantly with increasing histology grade (Fig. 1D), reminiscent of the above-mentioned association of Puf-A expression with tumor grade (Fig. 1A). These results prompted us to assess whether the abnormal p53 expression correlated with Puf-A expression. As shown, Puf-A expression was significantly higher in tumors with high p53 expression than those with low p53 expression (*p* < 0.001, Fig. 1D). We further used Pan-Cancer Atlas of TCGA to analyze the Puf-A RNA expression and TP53 status in patients with NSCLC and CRC. For both NSCLC (*p* = 0.023) and CRC (*p* = 0.003), *PUM3* level was significantly higher for patients with mutation *TP53* than those with no mutation *TP53* (Fig. S5).

We also examined the expression of Puf-A in colorectal cancer. Puf-A expression in the tumor part of colorectal cancers was elevated when compared to non-tumor part (Fig. 1E). Kaplan–Meier analysis showed that OS was higher for the patients with low expression of Puf-A compared to those with high expression of Puf-A (*p* = 0.004; Fig. 1E). This result was consistent with data mining results, which showed that high expression of Puf-A for patients with colorectal cancer significantly correlated with shorter OS of patients (*p* = 0.042; Fig. 1E).

### Puf-A expression is suppressed by p53

**Fig. 2:**
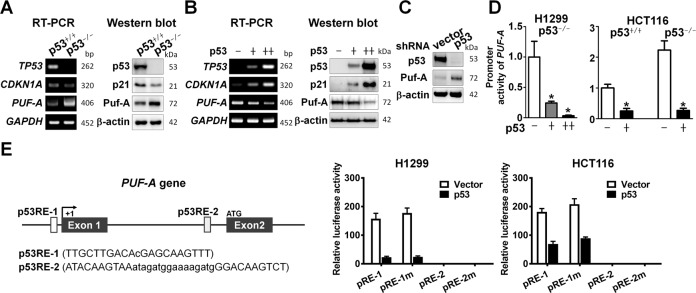
The expression of Puf-A is transcriptionally suppressed by p53. **A** RT-PCR and Western blot analyses of *PUF-A* (Puf-A) *TP53* (p53), and *CDKN1A* (p21) expression in p53^+/+^- and p53^−/−^-HCT116 cells were performed. GAPDH and β-actin were used, separately, as internal controls. **B** RT-PCR and Western blot. Analyses of *PUF-A* (Puf-A), *TP53* (p53), and *CDKN1A* (p21) expression in H1299 cells after transduction with control lentivirus (−), or low (+) and high (++) titers of p53-expressing lentivirus were performed. GAPDH andβ-actin were used, separately, as internal controls. **C** Western blot analysis of Puf-A in A549 cells transfected with control (shNC) or shp53-expressing vectors was performed. β-actin was used as an internal control. **D** The promoter activity of *PUF-A* in H1299 cells after transfection with control lentivirus (−), or low (+) and high (++) amounts of p53-expressing lentivirus was determined. *p < 0.05 (one-way ANOVA). Similarly, the promoter activity of *PUF-A* in p53^+/+^- and p53^−/−^-HCT116 cells after transfection with p53-expressing lentivirus (+) was determined. Mean ± SD values (*n* = 3) are shown. *p < 0.05 (two-tailed *t* test). **E** Two p53-response elements (p53RE-1 and p53RE-2) with the specific sequences are shown in the *PUF-A* gene. The pRE-1 and pRE-2 constructs drove by DNA fragment containing the p53-binding sites p53RE-1 (−720 to −700 nt) or p53RE-2 (+3497 to +3532 nt), respectively; the pRE-1m and pRE-2m constructs consisted of the region with mutations. These plasmids were transduced in H1299 and p53^+/+^ HCT116 cells. Normalized activity of luciferase reporter with a control plasmid was shown. Mean ± SD was presented.

The clinical observation of a correlation of Puf-A expression with p53 status of NSCLC tumors prompted us to examine the expression of Puf-A RNA and protein in a pair of p53^+/+^ and p53^−/−^ colorectal carcinoma cell line, HCT116. The expression of both Puf-A RNA and protein was indeed greater in p53^−/−^-HCT116 cells than those in the p53^+/+^-HCT116 cells, along with the opposite findings for p21 expression, as expected (Fig. 2A).

To investigate whether p53 influenced Puf-A expression, we transfected a p53^null^ NSCLC cell line, H1299, with the p53-expressing lentivirus at low and high titers. As shown in Fig. 2B, expression of p53 in H1299 cells reduced the expression of Puf-A RNA and protein in a dose-dependent manner, along with the increase of p53 downstream gene, CDKN1A, as expected. Similarly, p53 expression in p53^−/−^-HCT116 cells led to a decrease in Puf-A expression (Fig. S6). This was further corroborated by a significant increase in Puf-A expression upon p53 silencing in NSCLC cell lines A549 cells (Fig. 2C) and H460 (Fig. S6), both of which harbored wild-type TP53. Similar results were obtained using the colorectal cell lines p53^+/+^ HCT116 cells (Fig. S6).

Since the prevailing function of the p53 is the transcriptional control of target genes, we sought to delineate whether p53 could repress promoter activities of Puf-A gene. We cloned Puf-A regulatory control region (from -6508 to +5622) into a luciferase reporter vector. Upon transfection of the p53^−/−^H1299 cells with this reporter vector and the p53-expressing lentivirus, the promoter activity of *PUF-A* in these cells was markedly suppressed by the exogenously added p53 in a dose-dependent manner (Fig. 2D). Similarly, transfection of the paired p53^+/+^- and p53^−/−^-HCT116 cells with the reporter vector and the p53-expressing lentivirus showed that the presence of p53 also significantly reduced *PUF-A* promotor activity (Fig. 2D).

To further examine whether the apparent transcriptional repression of Puf-A by p53 could be attributed to direct binding of p53 to a specific regulatory region of Puf-A, we scanned the genomic sequences of Puf-A gene by ConTra V3 in order to identify putative high-affinity binding sites for p53 [21]. Two putative p53-response elements were identified in the Puf-A genome, −720 to −700 nt (p53RE-1) and +3497 to +3532 nt (p53RE-2), each containing two decamer motifs RRRCWWGYYY separated by a spacer, where W is A/T, R is C/G, and Y is C/T [22, 23] and the CWWG in the motif was shown to be a pre-requisite for high-affinity binding of p53 [24–26]. Thus, we generated pRE-1 and pRE-2 constructs by cloning the 791 bp (−739 to +51) and 808 bp (+3493 to +4300) fragments containing these putative p53-binding elements, which were then fused with SV40 promoter, and inserted into the pGL3 luciferase reporter vector. Meanwhile, another two luciferase reporter constructs with p53-response element mutation AWWA, pRE-1m and pRE-2m (which contained the mutated sequences as TTGATTAACAcGAGAAAATTT and ATAAAAATAAatagatggaaaagatgGGAAAAATCT, respectively), were also prepared. Transfection of p53^−/−^H1299 or HCT116 cells with pRE-1 led to the transcriptional activation of the luciferase reporter gene which was repressed following the expression of p53. However, transfection with the mutant construct, pRE-1m, the enhanced activity of the luciferase reporter gene was still suppressed by p53-expression (Fig. 2E). Besides, the transcriptional activity of the p53RE-2 was very low, regardless of the expression of p53 or not (Fig. 2E). These results implied that even though the downregulation of Puf-A was associated with p53 expression, p53 might not bind directly to the promoter region of Puf-A to regulate the transcription of the Puf-A gene.

Next, we used site-direct mutagenesis to generate three p53 hotspot mutants, R175H, R248W, and R273H, cloned them into HA-tag expression vector, and confirmed their protein expression by western blot with anti-HA antibody (Fig. S7). We used a luciferase reporter pp53-TA-luc, which contains p53 cis-acting element, to determine the transcriptional activity of the p53 mutants. The three p53 mutants exhibited no transactivation activity in H1299 and HCT116 p53^−/−^ cells as expected (Fig. S7). We used a luciferase reporter vector with Puf-A promoter region to address whether these p53 mutants regulate the expression of Puf-A. As shown in Supplementary Fig. S8, unlike wild-type p53, p53 hotspot mutants could not repress Puf-A promoter activity in H1299 and HCT116 p53^−/−^ cells. The three p53 mutants also fail to repress the reporter activity of p53-response element wild-type (pRE-1) and mutation (pRE-1m). Although the predicted p53-binding site of Puf-A promoter (RE1-m) was mutated, wild-type p53 could still repress Puf-A promoter activity. Besides, the p53-null H1299 cells were transfected with wild-type or p53 mutants. As shown in Supplementary Fig. S9, p53 and HA signals were detected in H1299 cells transfected with wild-type or mutant p53. However, only wild-type p53, but not p53 mutants, could inhibit Puf-A expression. These results indicated that repressed Puf-A expression by wild-type p53 is indirect but specific.

We also performed ChIP-q-PCR of p53-binding to the endogenous *PUM3* (Puf-A) and *CDKN1A* (P21) loci, which is a bona fide p53 target gene as a control, in A549 (p53 WT) and CL1-5 (p53R248W) cells. ChIP-q-PCR amplicons were designed to amplify the p53-response element regions of *PUM3* and *CDKN1A*. In A549 cells, we observed association of p53 with *PUM3* as well as *CDKN1A*. In contrast, this ChIP-q-PCR signals for *PUM3* and *CDKN1A* were decreased in CL1-5 cells (p53R248W) (Fig. S10). These data support the view that wild-type p53 can bind *PUM3*. However, protein and DNA interaction by ChIP assay does not necessarily infer regulation of gene expression. In fact, our reporter assay as described above suggests the repression of Puf-A by p53 is indirect but specific.

### Puf-A silencing in cancer cell lines impedes cell growth in vitro

**Fig. 3:**
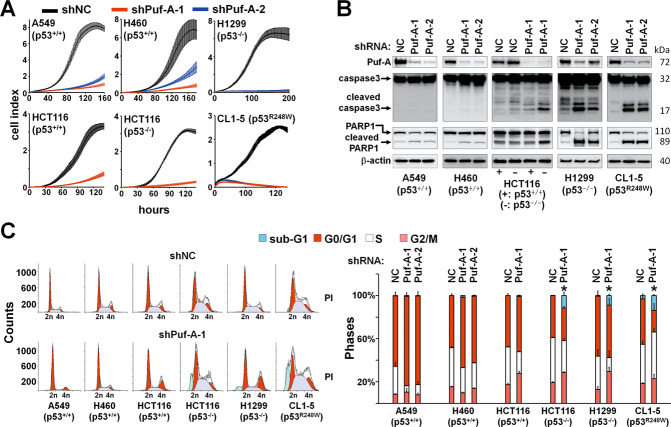
Silencing of Puf-A inhibits cancer proliferation in vitro. **A** Proliferation of various cancer cell lines over time, including p53^+/+^-A549, -H460, and -HCT116 cells and p53^−/−^-HCT116 and -H1299 cells, and p53^R248W^-CL1-5 cells, after transduction with control shNC, shPuf-A-1 (red line) and shPuf-A-2 (blue line). **B** Lentiviruses with shPuf-A-1 and -2 for silencing Puf-A were transduced into the indicated cancer cell lines. The expression levels of Puf-A, caspase 3, cleaved caspase 3, PARP1, cleaved PARP1, and β-actin were determined by western blot. **C** Flow cytometric analysis of cell-cycle distribution of Puf-A-silenced cells. All cell lines were infected with control shNC, shPuf-A-1, or -2 and then incubated with propidium iodide for assessment of cell-cycle distribution based on DNA content.

To understand the interplay between Puf-A and p53 in cancer cells, we suppressed Puf-A expression in p53^+/+^-A549, p53^+/+^-H460, p53^+/+^-HCT116, p53^−/−^-HCT116, p53^−/−^-H1299, and p53^R248W^-CL1-5 cells by infection with shPuf-A-1 or shPuf-A-2 lentivirus. After Puf-A silencing, the growth rates of all cancer cells were significantly reduced (Fig. 3A). To exclude an off-target knockdown, rescue experiment was performed. After treatment of 53^−/−^-H1299 cells with shPuf-A for 48 h, Puf-A was overexpressed in these cells, and proliferation of the cells was determined by AlamarBlue assay. The decreased proliferation of the Puf-A-silencing cells could be rescued by transfection with Puf-A-expressing plasmid (*p* < 0.0001; Fig. S11), supporting the specificity of shPuf-A for Puf-A expression. The proliferation of cells treated with shNC + Puf-A was greater than those treated with shNC + NC (*p* < 0.0001), consistent with the notion that Puf-A could promote growth of cancer cells. To further confirm the effect of Puf-A on cell proliferation, we determined the proliferation of p53^+/+^-A549 cells transfected with Puf-A- or control vectors. The proliferation of A549 cells was enhanced by Puf-A gene transfection in a dose- (control, 3104 ± 1482; 50 ng, 6824 ± 1243; and 100 ng, 10,294 ± 1076, *p* = 0.03 and 0.001, respectively) and time-dependent manner (48 h, 4423 ± 685.7 vs. 72 h, 10,076 ± 1267, *p* = 0.0024) (Fig. S12A, B).

To assess whether Puf-A affects cancer stemness of cancer cells, we transfected A549 cells with control and Puf-A-encoding plasmids and performed sphere formation assay. Puf-A overexpressing A549 cells had greater number and size of spheroids (11.3 ± 4.12) as compared to the mock cell cultures (6 ± 1.8, *p* = 0.002; Fig. S13A, B).

Given that apoptosis and cell-cycle arrest could contribute to growth inhibition, we also measured caspase 3 and Poly(ADP-ribose) polymerase 1 (PARP1) by western blot and performed cell-cycle analyses. Puf-A silencing in cells with normal p53 did not substantially raise the levels of cleaved caspase 3 or PARP1 (Fig. 3B). In contrast, Puf-A silencing in cells with abnormal p53 markedly increased cleaved caspase 3 and PARP1. Flow cytometry analyses revealed that Puf-A knockdown in cells with normal p53 increased the proportion of cells in the G0/G1 phase, along with a decrease in the S phase (Fig. 3C), indicating that Puf-A knockdown in cells with normal p53 induced cell-cycle arrest. On the other hand, Puf-A silencing in cells with abnormal p53 led to an increase in the sub-G1 phase (Fig. 3C), consistent with apoptosis.

### Puf-A plays an important role in a mouse model of mutant Kras/p53 loss driven lung ADC

**Fig. 4:**
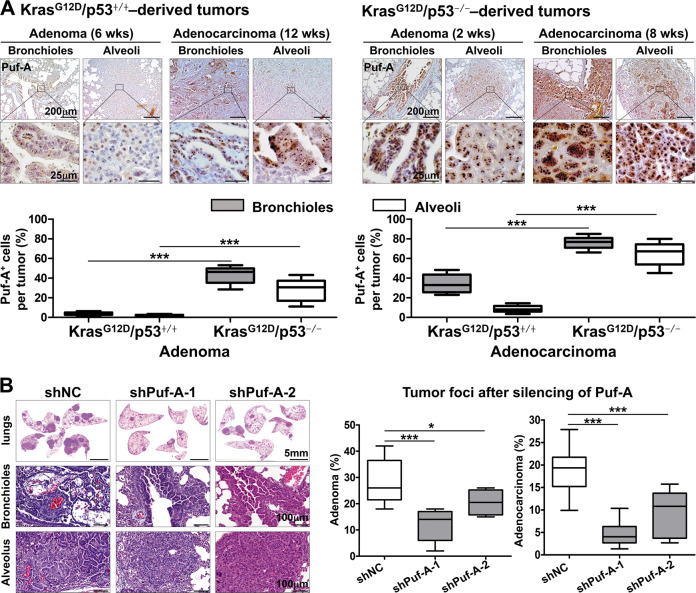
Silencing of Puf-A inhibits tumor progression in a spontaneous lung cancer mouse model. **A** Lung tissues with tumors from CCSP-rtTA/TetO-Cre/LSL-Kras^G12D^/p53^+/+^ mice after Kras^G12D^ activation for 6 and 12 weeks (left panel) and from CCSP-rtTA/TetO-Cre/LSL-Kras^G12D^/p53^flox/flox^ mice after Kras^G12D^ activation and p53 deletion for 2 weeks and 8 weeks (right panel) were respectively stained with the antibody against Puf-A. The enlarged images of Puf-A staining in the enclosed areas are shown below. Quantification of Puf-A^+^ cells in adenomas and ADCs derived from LSL-Kras^G12D^/p53^+/+^ (12 weeks old) and LSL-Kras^G12D^/p53^−/−^ mice (8 weeks old), respectively (bottom panel). ***p < 0.001 (one-way ANOVA). **B** Lentiviral vectors expressing shPuf-A-1, shPuf-A-2, or control shLacZ were intranasally delivered to the lungs of CCSP-rtTA/TetO-Cre/LSL-Kras^G12D^/p53^flox/flox^ mice four times between weeks 2 and 4. The areas of tumor involvement in the lungs were categorized as adenoma or ADC, measured by Metamorph and expressed as the percent of total lung involved at week 8 after Kras^G12D^ activation and p53 deletion. Box-whisker plots are shown for control shNC (*n* = 13), shPuf-A-1 (*n* = 11), and shPuf-A-2 (*n* = 6). *p < 0.05 and ***p < 0.001 (one-way ANOVA).

To confirm the in vivo interactions between Puf-A and p53, we employed a previously established lung cancer model using CCSP-rtTA/TetO-Cre/LSL-Kras^G12D^/p53^+/+^ mice [27], which developed lung adenoma, followed by ADC at 6 and 12 weeks, respectively, after Kras^G12D^ induction (Fig. 4A). Here we examined the in vivo impact of p53 on the expression of Puf-A and the development of lung tumors by generating CCSP-rtTA/TetO-Cre/LSL-Kras^G12D^/p53^flox/flox^ mice. As shown in Fig. 4A, these conditional p53^flox/flox^ mutant mice developed adenomas and ADC in the lungs as early as 2 weeks and 8 weeks, respectively, upon Kras^G12D^ activation, which were significantly accelerated compare to the p53^+/+^ mice. In addition, in Kras^G12D^/p53^+/+^ mice, only a few Puf-A^+^ cells (<5%) were found in bronchiolar and alveolar adenomas after 6 weeks of Kras^G12D^ induction. In contrast, in Kras^G12D^/p53^−/−^ mice, Puf-A^+^ cells were found in 44% and 28% in bronchiolar and alveolar adenomas, respectively, as early as 2 weeks (Fig. 4A). Furthermore, the percentage and the intensity of Puf-A expression in ADC were more prominent in these mice: the Puf-A^+^ cells increased from 30 and 7% in bronchiolar and alveolar ADC in Kras^G12D^/p53^+/+^ mice (12 weeks old) to 74 and 65% in Kras^G12D^/p53^−/−^ mice (8 weeks old) (Fig. 4A).

We then determined whether suppression of Puf-A expression after the onset of adenomas might prevent progression to ADCs in the lungs of CCSP-rtTA/TetO-Cre/LSL-Kras^G12D^/p53^flox/flox^ mice. Lentiviral vectors expressing shPuf-A-1, shPuf-A-2, or control shLacZ, were intranasally delivered to CCSP-rtTA/TetO-Cre/LSL-Kras^G12D^/p53^flox/flox^ mice four times between week 2 and week 4. The tumor foci in the lungs were examined at week 8 after Kras^G12D^ activation and p53 deletion. The percentage of lung tissue occupied by adenoma reduced from 26% in the mice treated with shLacZ, to 14% and 20% in mice treated with shPuf-A1 and shPuf-A2 lentivirus, respectively. In addition, ADC formation also decreased from 18% to 4% and 10%, respectively (Fig. 4B). Besides, the lung tissues were harvested to evaluate the proliferation and apoptosis in lung tumors, using Ki-67 staining and TUNEL assay, respectively. As shown in Supplementary Fig. S14, the frequency of Ki-67 positive cells in tumors of shPuf-A-treated mice was significantly lower than those treated with shLacZ. Moreover, compared to control mice, there was a significant increase in the frequency of TUNEL positive cells in tumor tissue of shPuf-A-treated mice. These data indicate that silencing Puf-A in vivo can not only inhibit tumor cell proliferation but also induce apoptosis of tumor cells. These results support a crucial role of Puf-A in the progression of NSCLC.

### Silencing of Puf-A reduces the activity of ribosome biogenesis

**Fig. 5:**
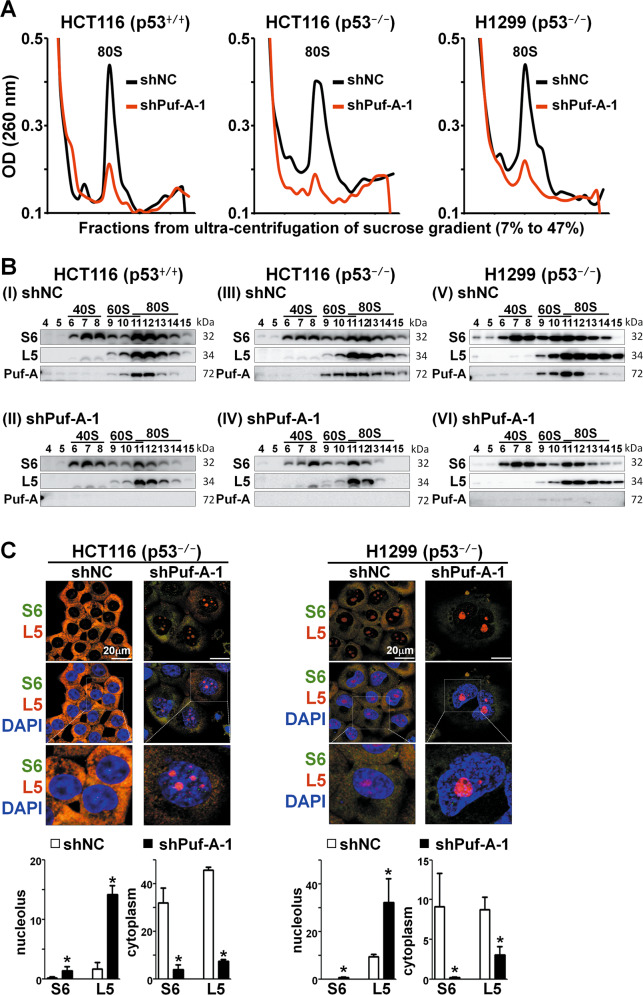
Puf-A silencing reduces ribosome biogenesis. **A** Polysome profiles of ribosome extracts from cancer cell lines (p53^+/+^-HCT116, p53^−/−^-HCT116 and p53^−/−^-H1299) that were transduced with control shNC (black) and shPuf-A-1 (red) lentivirus were analyzed. **B** Equal volumes of protein extracts of various ribosomal fractions from p53^+/+^-HCT116, p53^−/−^-HCT116 and p53^−/−^-H1299 cells after transduction were precipitated using trichloroacetic acid and then analyzed via western blot with antibodies against S6, L5, and Puf-A. (I, III, and V) with control shNC; and (II, VI, and VI) with shPuf-A-1. **C** Immunofluorescence staining of p53^+/+^-HCT116, p53^−/−^-HCT116, and -H1299 cells, were transduced, separately, with shNC (control) or shPuf-A-1 virus. These cells were then analyzed with antibodies against L5 (red) and S6 (green) and counterstained with DAPI (blue). The expression of S6 and L5 proteins in the cytoplasm and nucleolus from p53^+/+^-HCT116, p53^−/−^-HCT116, and -H1299 cells after transduction was quantized by ImageJ software.

IHC analysis of ADC and SCC displayed a distinctive Puf-A signal in the nucleolus of tumor cells (Fig. 1A). It is well known that the nucleolus, the subcellular region for ribosome biogenesis, tends to be more prominent in cancer than normal cells [28], and ribosome biogenesis is more robust in cancer cells. Besides, one of the PUF protein family, Puf6 in yeast, which shares ∼24% sequence identity with Puf-A, was shown to be involved in pre-rRNA processing [3]. To decipher whether Puf-A is involved in the ribosome biogenesis, we used sucrose gradient centrifugation to assess sedimentation patterns of ribosome biogenesis. We found that Puf-A silencing with shPuf-A-1 led to marked decreases in the 80S peak in p53^+/+^-HCT116, p53^−/−^-HCT116 and p53^−/−^-H1299 cells (Fig. 5A), especially in those cells with defective p53. Western blot analysis of gradient fractions revealed significant decreases in S6 (a marker for the 40S ribosome) [29] and L5 (a marker for the 60S ribosome) [30] upon Puf-A silencing (Fig. 5B II, IV, and VI), as compared to cells treated with control shNC (Fig. 5B I, III, and V). The subcellular localization of S6 and L5 proteins in response to Puf-A silencing was further examined by immunofluorescence microscopy (Fig. 5C). Upon Puf-A silencing in p53^−/−^-HCT116 and p53^−/−^-H1299 cells by shPuf-A-1 lentivirus, the expression of S6 and L5 markedly diminished in the cytoplasm along with accumulation in the nucleolus. To further address this issue, we performed additional experiments to examine the expression of NPM1, ribosomal protein L5 (RPL5), and Puf-A in clinical NSCLC specimens with high and low expression of Puf-A, using Opal multiplex immunofluorescence assay. In samples with low Puf-A expression, we found NPM1 to be dispersed in the nucleoplasm and RPL5 to be markedly diminished in the cytoplasm but present in the nucleolus. In contrast, in samples with high Puf-A expression, we observed co-localization of Puf-A and NPM1 in the nucleolus and the distribution of RPL5 in the cytoplasm (Fig. S15). These results support that Puf-A plays an important role in ribosome biogenesis.

### Puf-A interacts with NPM1 in nucleolus

**Fig. 6:**
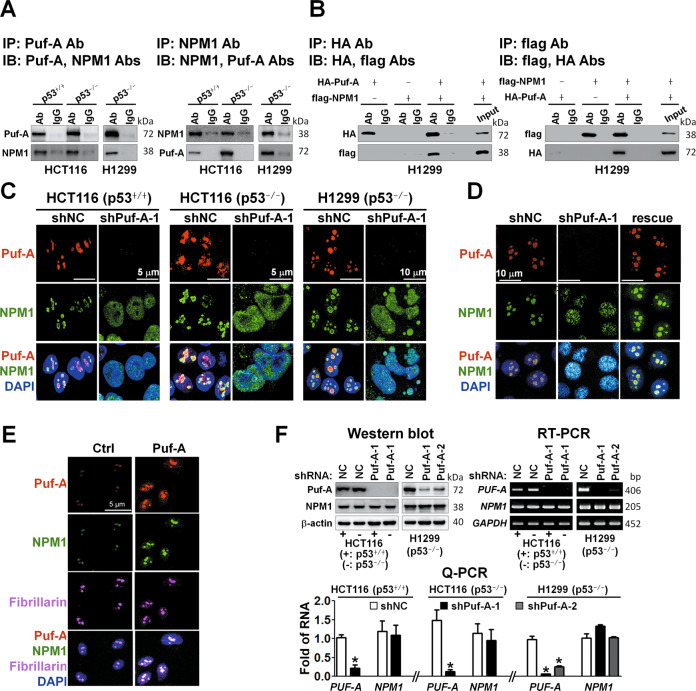
Puf-A interacts with NPM1 in nucleolus. **A** Co-immunoprecipitation (Co-IP) of endogenous Puf-A and NPM1 proteins. Cell lysates from p53^+/+^HCT116, p53^−/−^HCT116 and H1299 cells were co-IP with antibodies directed against Puf-A or NPM1 and subsequently immunoblotted (IB) with Puf-A and NPM1 antibodies as indicated. **B** Co-IP of exogenous Puf-A and NPM1 proteins. Cell lysates obtained from H1299 cells after co-transfection with HA-tagged Puf-A (HA-Puf-A) and flag-tagged NPM1 (flag-NPM1) overexpression vector, were co-IP with antibodies directed against HA or flag, and subsequently immunoblotted with tag antibodies as indicated. **C** Immunofluorescence staining for Puf-A and NPM1 expression in p53^+/+^- and p53^−/−^-HCT116 cells and p53^−/−^-H1299 cells. The cells were transduced with control shNC and shPuf-A-1 and stained with antibodies directed against Puf-A (red) and NPM1 (green) and counterstained with DAPI (blue). **D** Rescue experiments. H1299 cells were transfected with control (shNC) and shPuf-A-1 plasmids. After 48 h, the shPuf-A-1-transfected H1299 cells were transfected with 100 ng control and Puf-A-expressing plasmids. Three days later, NPM localization was determined by an immunofluorescence assay. **E** Overexpression of Puf-A in A549 cells. Representative images of Puf-A (red), NPM1 (green), and fibrillarin (purple) immunofluorescence in A549 cells expressing control (NC) or Puf-A plasmids with DAPI nuclear stain (blue). Scale bar, 5 μm. **F** Western blot, RT-PCR and Q-PCR analyses of the expression of Puf-A (*PUF-A*) and NPM1 (*NPM1*) in p53^+/+^-, p53^−/−^-HCT116 and p53^−/−^-H1299 cells after transduction with shPuf-A-1, shPuf-A-2, or control shNC. β-actin and GAPDH were used as internal controls. In Q-PCR, mean ± SD values (*n* = 3) are shown. * refers to *p* < 0.05 (one-way ANOVA).

Another key player in ribosome biogenesis is nucleophosmin (NPM1) [31]. Thus, we investigated the possible interplays between Puf-A and NPM1. Co-immunoprecipitation of Puf-A and NPM1 were demonstrated in p53^+/+^HCT116, p53^−/−^HCT116, and H1299 cells (Fig. 6A). The mass spectrometric analyses of the proteins immunoprecipitated by Puf-A antibody revealed NPM1 as one of the Puf-A-interacting proteins (Fig. S16). In order to verify their interactions, we overexpressed HA-tagged Puf-A and flag-tagged NPM1 in H1299 cells and showed that HA-tagged Puf-A co-precipitated with flag-tagged NPM1 and vice versa (Fig. 6B).

It’s been shown that NPM1 could interact with nucleolar proteins containing arginine-rich linear motifs, such as GNL2 and SURF6 [32]. Using protein–protein docking program GRAMM-X, we predict CPL domain of Puf-A as a region for binding to NPM1, which contains ^478^RRR^480^ (Fig. S17A). Therefore, Puf-A was mutated from the ^478^RRR^480^ to ^478^GGG^480^ and cloned into an expression vector containing a HA tag. We overexpressed flag-tagged NPM1 along with HA-tagged Puf-A or Puf-A mutant in H1299 cells. As shown in Supplementary Fig. S17B, while wild-type HA-tagged Puf-A co-precipitated with flag-tagged NPM1, consistent with Fig. 6B, the mutant Puf-A did not co-precipitate with NPM1. To our knowledge, this is the first evidence showing that the residues ^478^RRR^480^ of Puf-A are important for binding of Puf-A to NPM1. We further performed a rescue experiment to evaluate whether a Puf-A mutant deficient in binding to NPM1 can rescue Puf-A-silenced H1299 cells. As shown in Fig. S11, unlike wild-type Puf-A (shPuf-A + Puf-A), mutant Puf-A (shPuf-A + Puf-A-mut) could not rescue the proliferation of Puf-A-silenced cells (*p* < 0.0001). This result indicates that the binding of Puf-A to NPM1 is important for cell proliferation.

Immunofluorescence staining indicated that the co-localization of Puf-A and NPM1 in the nucleolus (Fig. 6C, shNC). Interestingly, in cells treated with shPuf-A-1, NPM1 was largely dispersed to the nucleoplasm (Fig. 6C), although Puf-A silencing did not alter the expression of NPM1 protein or RNA (Fig. 6F) as compared to control cells. Furthermore, we overexpressed Puf-A with or without silencing of NPM1 in HCT116 (p53 WT) cells and used sucrose gradient centrifugation to evaluate whether the interaction between Puf-A and NPM1 affects ribosome biogenesis. As shown in Supplementary Fig. S18, the signals of 40S, 60S, and 80S were strikingly reduced in Puf-A-overexpressing cells with NPM1-silencing when compared to Puf-A-overexpressing cells without NPM1-silencing. This result demonstrates that interaction between Puf-A and NPM1 is important for ribosome biogenesis.

To confirm that disruption of NPM1 localization in nucleolus by shPuf-A was attributed to Puf-A silencing, a rescue experiment was performed. As shown in Fig. 6D, the exogenously expressed Puf-A construct successfully re-localized NPM1 to nucleolus in the Puf-A knockdown H1299 cells. Furthermore, overexpression of Puf-A in A549 cells led to detection of strong signals of NPM1 as well as Puf-A in the nucleolus by immunofluorescence (Fig. 6E), in contrast to weak expression of Puf-A and NPM1 in the nucleolus of cells transfected with control plasmid. To further confirm the location of Puf-A and NPM1, we stained fibrillarin, which is a nucleolus maker. Co-localization of Puf-A, NPM1, and fibrillarin at the nucleolus following Puf-A transfection of the A549 cells was observed (Fig. 6E), suggesting that Puf-A overexpression could not alter the distribution of NPM1 in the nucleolus. These data implied that Puf-A expression might dictate nucleolar localization of NPM1 through their direct interaction.

## Discussion

The clinical relevance of Puf-A expression has not been investigated except for a single report showing greater Puf-A expression in invasive breast cancer than ductal carcinoma in situ, but its expression level did not correlate with histology grade (*p* = 0.238), and its impact on clinical outcome was not addressed [5]. Here we showed that expression of Puf-A protein was greater in non-small cell lung cancer (NSCLCs), especially those with altered p53 expression, than normal lung tissues, and the expression level was not only positively correlated with histology grade but also highly prognostic for stage I NSCLC. To the best of our knowledge, this is the first study demonstrating the prognostic impact of Puf-A in cancer. Since ~1 in 5 patients with completely resected early-stage NSCLC will recur within 2 years [33], it is imperative to identify risk factors and biomarkers predictive of recurrence. In spite of more than 500 reported studies for prognostic evaluation, not a single protein marker has yet been validated sufficiently for clinical use [34]. Thus, our finding of Puf-A as a prognostic marker for stage I NSCLC may address this unmet need, if verified in further studies.

The clinical significance of Puf-A expression suggests a critical role of Puf-A in tumor progression. Indeed, Puf-A silencing inhibited the growth of lung and colorectal cancer cells in vitro and development of Kras^G12D^-driven lung tumors in vivo. Furthermore, we observed higher Puf-A expression in Ki-67^high^ tumor tissues than in Ki-67^low^ tumor tissues, and overexpression of Puf-A increased cell proliferation. This is consistent with the report that Puf-A overexpression increased colony forming ability of MDA-MB231 breast cancer cells and their growth in nude mice [5]. These data suggests that Puf-A may play an important role in tumorigenesis.

Association of Puf-A expression with abnormal p53 expression suggested possible transcriptional regulation of Puf-A by p53. However, our reporter assays suggested that p53 might regulate the transcription of Puf-A indirectly. Recent advances in p53 function also indicate that p53 could indirectly downregulate gene expression through p21, which is a cyclin dependent kinase inhibitor [35]. p21, a direct p53 target, stabilizes RB (retinoblastoma protein)-related DREAM (DP, RB-like, E2F4 and MuvB) complex to downregulate cell-cycle genes, such as BIRC5, CDC25C, and PLK1 [36, 37]. These genes that were downregulated by p53 were not reduced in doxorubicin-treated p21-null cells by RNA-seq analysis [37], suggesting p53 might not directly regress the transcription of these genes [38, 39]. In this study, we found upregulation of p21 in p53-overexpressing cells. Whether p21 is involved in the transcriptional downregulation of Puf-A by p53 awaits further investigation.

Our observation of the growth inhibition by silencing Puf-A in vitro in various cancer cell lines, especially in those with abnormal p53 expression, suggests that Puf-A might be an attractive therapeutic target for cancer with abnormal p53. A reduction in Kras^G12D^-driven tumor foci by administration of shPuf-A in p53-deficient mice further reinforced this notion. Besides, Puf-A silencing also suppressed the growth of cancer cells with normal p53, indicating that Puf-A might play a decisive role in regulating tumor cell growth, even when its expression was restricted by p53.

Genotoxic stress can cause disruption of ribosome biogenesis. In this context, L5, L11, and L23 ribosomal proteins associate with HDM2 to abrogate ubiquitination of p53, leading to p53 activation [40–42]. Interestingly, we found that Puf-A silencing decreased ribosomal biogenesis, regardless of p53 status. We think that wild-type p53 might indirectly inhibit Puf-A expression to decrease ribosome biogenesis. While p53 is mutated, it could not inhibit Puf-A expression. Then Puf-A might maintain or increase ribosome biogenesis and exhibit anti-apoptosis activity to promote tumorigenesis.

The observation of predominant nucleolar localization of Puf-A is intriguing. The nucleolus is the nuclear subdomain with no membrane. It can be segregated into three compartments, including fibrillary center, dense fibrillary component, and granular component [43]. NPM1 is found to reside in the granular component of nucleolus [44]. It is likely that Puf-A might also be located in the granular component of nucleolus when it binds to NPM1. Indeed, we showed that Puf-A directly interacted with NPM1 by immunoprecipitation with specific antibodies. It was reported that the acidic tracts of NPM1 could interact with other nucleolar proteins which contain arginine-rich linear motifs [32]. Sequence analysis of the Puf-A reveals that Puf-A contains two arginine-rich motifs in residues 146-147 and 478–480, suggesting that Puf-A might directly interact with NPM1 through these two arginine-rich motifs. We demonstrated that the ^478^RRR^480^ residues of Puf-A was important for binding to NPM1.

NPM1 is involved in tumor progression, especially with the role in ribosome biogenesis that is increased in cancer cells [45, 46]. It promotes the nuclear export of 40S and 60S ribosomal subunits into the cytoplasm to increase the number of 80S ribosomes, and then leading to increased cell proliferation [47]. The over-activated ribosome biogenesis is consistent with the concept that cancer cells require extensive protein synthesis, which relies on a constant supply of new ribosomes [48]. We found that the ribosome biogenesis was disrupted and NPM1 dispersed in the nucleoplasm in Puf-A-silenced cancer cells. These data suggest that Puf-A might control the location of NPM1 to regulate the processes of nuclear export of ribosomal subunits.

In summary, Puf-A plays an important role in cancer progression for NSCLC and CRC. Combined with the prognostic value of Puf-A in NSCLC observed in this study, development of Puf-A-targeted cancer theranostics is warranted.

## Materials and methods

### Tumor sections of human cancers

Tumor sections of 82 patients with NSCLC were obtained from the tissue bank of Chang Gung Memorial Hospital (CGMH) at Linko under the approved of the Institutional Review Board (IRB) at CGMH (IRB #103-3940C). Informed consent was obtained from all subjects and the experiments conformed to the principles set out in the WMA Declaration of Helsinki and the Department of Health and Human Services Belmont Report. Clinical characteristics of the NSCLC patients are shown in Table S1. Two CRC tissue microarrays (CDA3 and CD4) were purchased from SuperBioChips (Seoul, Korea).

### Immunohistochemical staining

Formalin-fixed paraffin sections were incubated overnight with antibodies against Puf-A, p53 (NCL-P53-DO7, Novocastra, Leica Biosystems, UK), and Ki-67 (NCL-L-Ki-67-MM1, Leica Biosystems). After washing, tissue slides were incubated with biotinylated secondary antibodies for 1 h at room temperature, and then incubated with horseradish peroxidase-conjugated streptavidin (Vector, USA). All sections were counterstained with Mayer’s hematoxylin. Puf-A, p53, and Ki-67 stained tissue sections were read by the pathologist YLH and evaluated with a histological scoring system, H-score [49]. Ki-67 was low if there is <14% of nuclei staining and high if ≥14% [50, 51]. The Youden index, which was calculated from the receiver operating characteristic curve, was used to determine the optimal cut-off value for high versus low expression level.

### Cell culture

293T, A549, H460, and H1299 cells were obtained from the American Type Culture Collection (USA) (Authenticated Nov, 2018 by STR sequencing). p53^+/+^- and p53^−/−^-HCT116 cells were kindly provided by Dr. Yu-Ju Chen (Institute of Chemistry, Academia Sinica, Taiwan). CL1-5 cells were kindly provided by Dr. Pan-Chyr Yang (Graduate Institute of Oncology, National Taiwan University Medical College, Taiwan). 293 T, A549, H460, p53^+/+^- and p53^−/−^-HCT116 cells were cultured in 10% FBS/DMEM (Gibco, NY, USA); H1299 and CL1-5 cells were cultured in 10% FBS/RPMI-1640 (Gibco). Cells were passaged every 3 days in a 0.5% trypsin-EDTA solution and maintained until used.

### Conditional mutant mice

CCSP-rtTA and TetO-Cre transgenic mice and p53^flox/flox^ knock-in mice were obtained from the Jackson Laboratory (USA). LSL-Kras^G12D^/p53^+/+^ knock-in mice were obtained from the NCI Mouse Models of Human Cancers Consortium. All mice were housed in a pathogen-free environment, and experiments were performed with the approval of the Institutional Animal Care and Use Committee (IACUC) of Chang Gung University (IACUC #CGU-14-021). CCSP-rtTA/TetO-Cre/LSL-Kras^G12D^ conditional mutant mice were generated by crossing CCSP-rtTA/TetO-Cre transgenic mice and LSL-Kras^G12D^ knock-in mice. CCSP-rtTA/TetO-Cre/LSL-Kras^G12D^/p53^flox/flox^ conditional mutant mice were generated by crossing CCSP-rtTA/TetO-Cre /LSL-Kras^G12D^ conditional mutant mice and p53^flox/flox^ knock-in mice. Conditional mutant mice (6–8-week-old) were treated with doxycycline (1 mg/mL, Sigma-Aldrich, USA) in their drinking water to induce lung tumors.

### Immunofluorescence staining

The primary antibodies used for immunofluorescence staining were Puf-A (monoclonal antibody), NPM1 (sc-5564, Santa Cruz, TX, USA), S6 (sc-74459, Santa Cruz), L5 (ab157099, Abcam), fibrillarin (sc-374022, Santa Cruz), and fibrillarin (sc-374022, Santa Cruz). The secondary antibodies used for immunofluorescence staining were Alexa488-conjugated donkey anti-rabbit IgG, donkey anti-mouse IgG (Invitrogen, USA), Alexa555-conjugated donkey anti-mouse IgG, donkey anti-rabbit IgG (Jackson ImmunoResearch, PA, USA), and Alexa633-conjugated streptavidin. Briefly, cells were fixed in 4% paraformaldehyde/phosphate-buffered saline (PBS) for 20 min at room temperature, permeabilized with 0.5% Triton X-100 in PBS for 5 min, and then blocked with 5% bovine serum albumin/PBS for 30 min. Slides were incubated at 4 °C with primary antibodies. After overnight incubation, cells were washed and incubated for 1 h at room temperature with secondary antibodies. Cells were then counterstained with DAPI (Pharmingen, NJ, USA). The staining intensity of S6 and L5 protein in the cytosol and nuclei of cells was determined using Metamorph^TM^ software (Molecular Devices, USA).

### Statistical analysis

Statistical analysis was performed with Prism (GraphPad Software). One-way ANOVA and Student’s *t* test (two-tailed) were used to analyze the results of the experiments as indicated in the figure legend, and a *p* value < 0.05 was considered statistically significant. Data are expressed as the mean ± standard error. The Kaplan–Meier method was used to calculate OS, and differences between the groups were assessed by the log-rank test. The survival analysis of high and low expression of Puf-A in stage I NSCLC was analyzed using “survival” and “survminer” packages in R version 3.4.1.

Other methods, including plasmid construction, q-PCR, western blot, cell-cycle analysis, cell proliferation, co-immunoprecipitation, genotyping, intranasal delivery of lentivirus, PUF-A promoter activity, sucrose gradient fractionation, and primer sequences, are found in Supplementary Information.

## Supplementary information

Supplemental material

## Data Availability

All data generated or analyzed during this study are included in this published article and its Supplementary Information Files.

## References

[1] Kuo MW, Wang SH, Chang JC, Chang CH, Huang LJ, Lin HH (2009). A novel puf-A gene predicted from evolutionary analysis is involved in the development of eyes and primordial germ-cells. PloS ONE.

[2] Wang X, McLachlan J, Zamore PD, Hall TM (2002). Modular recognition of RNA by a human pumilio-homology domain. Cell..

[3] Qiu C, McCann KL, Wine RN, Baserga SJ, Hall TM (2014). A divergent Pumilio repeat protein family for pre-rRNA processing and mRNA localization. Proc Natl Acad Sci USA.

[4] Li Z, Lee I, Moradi E, Hung NJ, Johnson AW, Marcotte EM (2009). Rational extension of the ribosome biogenesis pathway using network-guided genetics. PLoS Biol.

[5] Fan CC, Lee LY, Yu MY, Tzen CY, Chou C, Chang MS (2013). Upregulated hPuf-A promotes breast cancer tumorigenesis. Tumour Biol.

[6] Stepinski D (2018). The nucleolus, an ally, and an enemy of cancer cells. Histochem Cell Biol.

[7] Schmidt-Zachmann MS, Hugle-Dorr B, Franke WW (1987). A constitutive nucleolar protein identified as a member of the nucleoplasmin family. EMBO J.

[8] Chen S, Meng T, Zheng X, Cai J, Zhang W, You H (2018). Contribution of nucleophosmin overexpression to multidrug resistance in breast carcinoma. J Drug Target.

[9] Li S, Zhang X, Zhou Z, Huang Z, Liu L, Huang Z (2017). Downregulation of nucleophosmin expression inhibited proliferation and induced apoptosis in salivary gland adenoid cystic carcinoma. J Oral Pathol Med.

[10] Sawazaki H, Ito K, Asano T, Kuroda K, Sato A, Asakuma J (2017). Increased nucleophosmin expression is a strong predictor of recurrence and prognosis in patients with N0M0 upper tract urothelial carcinoma undergoing radical nephroureterectomy. World J Urol.

[11] Li Z, Boone D, Hann SR (2008). Nucleophosmin interacts directly with c-Myc and controls c-Myc-induced hyperproliferation and transformation. Proc Natl Acad Sci USA.

[12] Liu GY, Shi JX, Shi SL, Liu F, Rui G, Li X (2017). Nucleophosmin regulates intracellular oxidative stress homeostasis via antioxidant PRDX6. J Cell Biochem.

[13] Chang HY, Fan CC, Chu PC, Hong BE, Lee HJ, Chang MS (2011). hPuf-A/KIAA0020 modulates PARP-1 cleavage upon genotoxic stress. Cancer Res.

[14] Kandoth C, McLellan MD, Vandin F, Ye K, Niu B, Lu C (2013). Mutational landscape and significance across 12 major cancer types. Nature..

[15] Kubbutat MH, Jones SN, Vousden KH (1997). Regulation of p53 stability by Mdm2. Nature..

[16] Haupt Y, Maya R, Kazaz A, Oren M (1997). Mdm2 promotes the rapid degradation of p53. Nature..

[17] Sionov RV, Haupt Y (1999). The cellular response to p53: the decision between life and death. Oncogene..

[18] Vousden KH, Lu X (2002). Live or let die: the cell’s response to p53. Nat Rev Cancer.

[19] Yang K, Wang M, Zhao Y, Sun X, Yang Y, Li X (2016). A redox mechanism underlying nucleolar stress sensing by nucleophosmin. Nat Commun.

[20] Xie D, Lan L, Huang K, Chen L, Xu C, Wang R (2014). Association of p53/p21 expression and cigarette smoking with tumor progression and poor prognosis in non-small cell lung cancer patients. Oncol Rep.

[21] Kreft L, Soete A, Hulpiau P, Botzki A, Saeys Y, De Bleser P (2017). ConTra v3: a tool to identify transcription factor binding sites across species, update 2017. Nucleic Acids Res.

[22] el-Deiry WS, Kern SE, Pietenpol JA, Kinzler KW, Vogelstein B (1992). Definition of a consensus binding site for p53. Nat Genet.

[23] Funk WD, Pak DT, Karas RH, Wright WE, Shay JW (1992). A transcriptionally active DNA-binding site for human p53 protein complexes. Mol Cell Biol.

[24] Inga A, Storici F, Darden TA, Resnick MA (2002). Differential transactivation by the p53 transcription factor is highly dependent on p53 level and promoter target sequence. Mol Cell Biol.

[25] Jordan JJ, Menendez D, Inga A, Noureddine M, Bell DA, Resnick MA (2008). Noncanonical DNA motifs as transactivation targets by wild type and mutant p53. PLoS Genet.

[26] Osada M, Park HL, Nagakawa Y, Yamashita K, Fomenkov A, Kim MS (2005). Differential recognition of response elements determines target gene specificity for p53 and p63. Mol Cell Biol.

[27] Cho HC, Lai CY, Shao LE, Yu J (2011). Identification of tumorigenic cells in Kras(G12D)-induced lung adenocarcinoma. Cancer Res.

[28] Montanaro L, Trere D, Derenzini M (2008). Nucleolus, ribosomes, and cancer. Am J Pathol.

[29] Volarevic S, Stewart MJ, Ledermann B, Zilberman F, Terracciano L, Montini E (2000). Proliferation, but not growth, blocked by conditional deletion of 40S ribosomal protein S6. Science.

[30] Rosorius O, Fries B, Stauber RH, Hirschmann N, Bevec D, Hauber J (2000). Human ribosomal protein L5 contains defined nuclear localization and export signals. J Biol Chem.

[31] Lindstrom MS (2011). NPM1/B23: a multifunctional chaperone in ribosome biogenesis and chromatin remodeling. Biochem Res Int.

[32] Mitrea DM, Cika JA, Guy CS, Ban D, Banerjee PR, Stanley CB, et al. Nucleophosmin integrates within the nucleolus via multi-modal interactions with proteins displaying R-rich linear motifs and rRNA. Elife. 2016;5:e13571. 10.7554/eLife.13571 PMC4786410 26836305

[33] Kiankhooy A, Taylor MD, LaPar DJ, Isbell JM, Lau CL, Kozower BD (2014). Predictors of early recurrence for node-negative t1 to t2b non-small cell lung cancer. Ann Thorac Surg.

[34] Zhu CQ, Tsao MS (2014). Prognostic markers in lung cancer: is it ready for prime time?. Transl Lung Cancer Res.

[35] Fischer M (2017). Census and evaluation of p53 target genes. Oncogene..

[36] Fischer M, Quaas M, Steiner L, Engeland K (2016). The p53-p21-DREAM-CDE/CHR pathway regulates G2/M cell cycle genes. Nucleic Acids Res.

[37] Fischer M, Quaas M, Nickel A, Engeland K (2015). Indirect p53-dependent transcriptional repression of Survivin, CDC25C, and PLK1 genes requires the cyclin-dependent kinase inhibitor p21/CDKN1A and CDE/CHR promoter sites binding the DREAM complex. Oncotarget..

[38] Fischer M (2016). p21 governs p53’s repressive side. Cell Cycle.

[39] Fischer M, Grossmann P, Padi M, DeCaprio JA (2016). Integration of TP53, DREAM, MMB-FOXM1 and RB-E2F target gene analyses identifies cell cycle gene regulatory networks. Nucleic Acids Res.

[40] Pestov DG, Strezoska Z, Lau LF (2001). Evidence of p53-dependent cross-talk between ribosome biogenesis and the cell cycle: effects of nucleolar protein Bop1 on G(1)/S transition. Mol Cell Biol.

[41] Golomb L, Volarevic S, Oren M (2014). p53 and ribosome biogenesis stress: the essentials. FEBS Lett.

[42] Bursac S, Brdovcak MC, Donati G, Volarevic S (2014). Activation of the tumor suppressor p53 upon impairment of ribosome biogenesis. Biochim Biophys Acta.

[43] Boisvert FM, van Koningsbruggen S, Navascues J, Lamond AI (2007). The multifunctional nucleolus. Nat Rev Mol Cell Biol.

[44] Ando K, Parsons MJ, Shah RB, Charendoff CI, Paris SL, Liu PH (2017). NPM1 directs PIDDosome-dependent caspase-2 activation in the nucleolus. J Cell Biol.

[45] Colombo E, Alcalay M, Pelicci PG (2011). Nucleophosmin and its complex network: a possible therapeutic target in hematological diseases. Oncogene..

[46] Ponkratova DA, Lushnikova AA (2020). The role of nucleophosmin in cell functioning and tumor progression. Biol Bull Rev.

[47] Maggi LB, Kuchenruether M, Dadey DY, Schwope RM, Grisendi S, Townsend RR (2008). Nucleophosmin serves as a rate-limiting nuclear export chaperone for the Mammalian ribosome. Mol Cell Biol.

[48] Dez C, Tollervey D (2004). Ribosome synthesis meets the cell cycle. Curr Opin Microbiol.

[49] Ishibashi H, Suzuki T, Suzuki S, Moriya T, Kaneko C, Takizawa T (2003). Sex steroid hormone receptors in human thymoma. J Clin Endocrinol Metab.

[50] Sinn HP, Schneeweiss A, Keller M, Schlombs K, Laible M, Seitz J (2017). Comparison of immunohistochemistry with PCR for assessment of ER, PR, and Ki-67 and prediction of pathological complete response in breast cancer. BMC Cancer.

[51] Iqbal N, Iqbal N (2014). Human epidermal growth factor receptor 2 (HER2) in cancers: overexpression and therapeutic implications. Mol Biol Int.

