# Supplementary material for: Puf-A promotes cancer progression by interacting with nucleophosmin in nucleolus
Source: Oncogene. 2022 Jan 9;41(8):1155–65. doi: 10.1038/s41388-021-02138-0 (PMC8856959; doi:10.1038/s41388-021-02138-0)
Supplement: Supplementary file 1 — Supplemental material [file 41388_2021_2138_MOESM1_ESM.docx]

**Supplementary Information**

**Puf-A interacts with nucleophosmin in nucleolus and promotes cancer progression**

Huan-Chieh Cho, Yenlin Huang, Jung-Tung Hung, Tsai-Hsien Hung, Kai-Chun Cheng, Yun-Hen Liu, Ming-Wei Kuo, Sheng-Hung Wang, Alice L. Yu, and John Yu

**I. Supplementary Figures**

**Figure S1**

**Figure S1. Data mining of the expression of *PUF-A* RNA in lung cancers.** A dataset GSE68571 showed the expression level of *PUF-A* RNA in patients with different grade. Mean values are indicated with red lines. ** *p* < 0.01 and *** *p* < 0.001 (one-way ANOVA with Dunn’s multiple comparisons test).

**Figure S2**


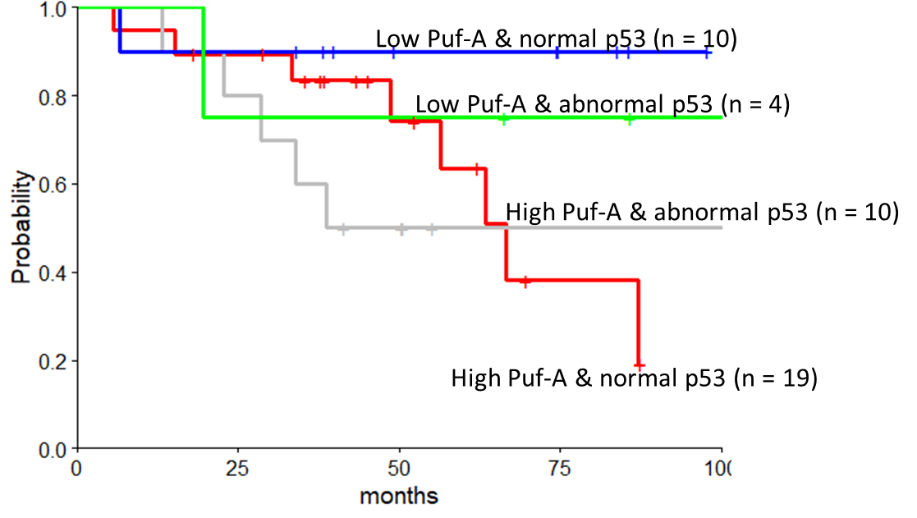


**Figure S2. Overall survival for stage I NSCLC stratified by Puf-A and p53 as determined by IHC.**

**Figure S3**

**
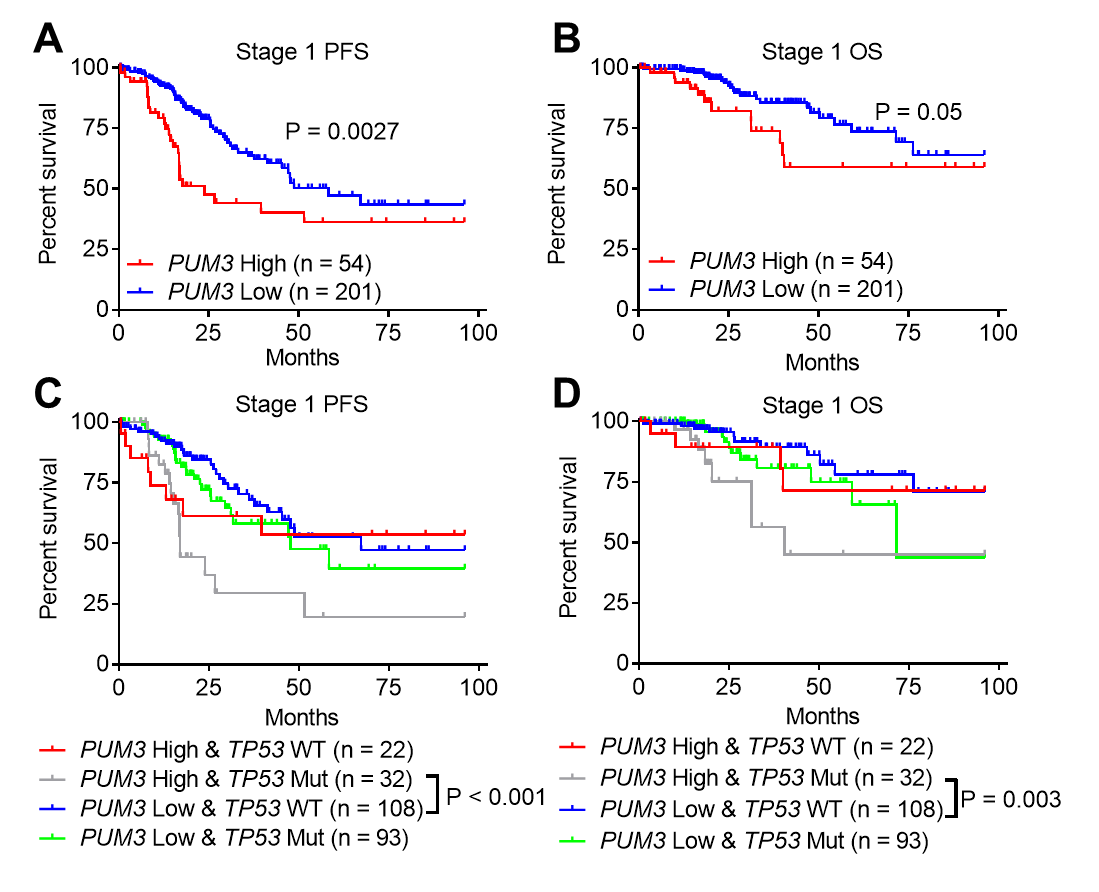
**

**Figure S3. The influence of *PUM3* level and *TP53* status on PFS and OS in patient with stage 1 NSCLC. TCGA Pan-cancer atlas was used for the analysis.** Kaplan-Meier curves showing RFS (A and C) and OS (B and D) of sage 1 NSCLC patients according to *PUM3* expression (A and B) or *PUM3* expression and *TP53* mutation status (C and D). Log-rank test was applied for statistical comparison.

**Figure S4**

**
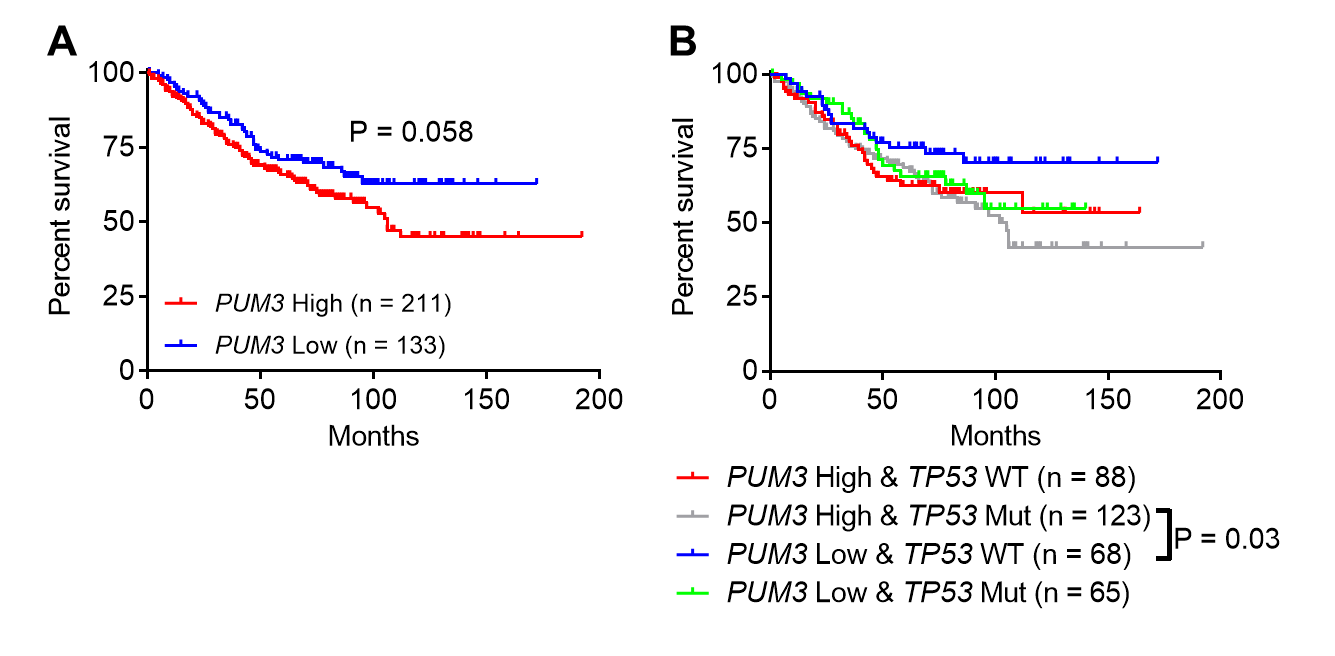
**

**Figure S4. The impact of *PUM3* level and *TP53* status on PFS and OS in patient with CRC.** GSE39582 database was used for the analysis. Kaplan-Meier curves showing OS of CRC patients according to *PUM3* expression (A) or *PUM3* expression and *TP53* mutation status (B). Log-rank test was applied for statistical comparison.

**Figure S5**

**Figure S5. Difference in gene expression level of *PUM3* (Puf-A) for patients with mutation and no mutation *TP53*.** NSCLC and CRC from the Pan-Cancer atlas of TCGA was used. RNA expression levels of Puf-A were presented as RNA-Seq by expectation-maximization (RESM). Two-tailed Mann-Whitney test was used for statistical comparison.

**Figure S6**


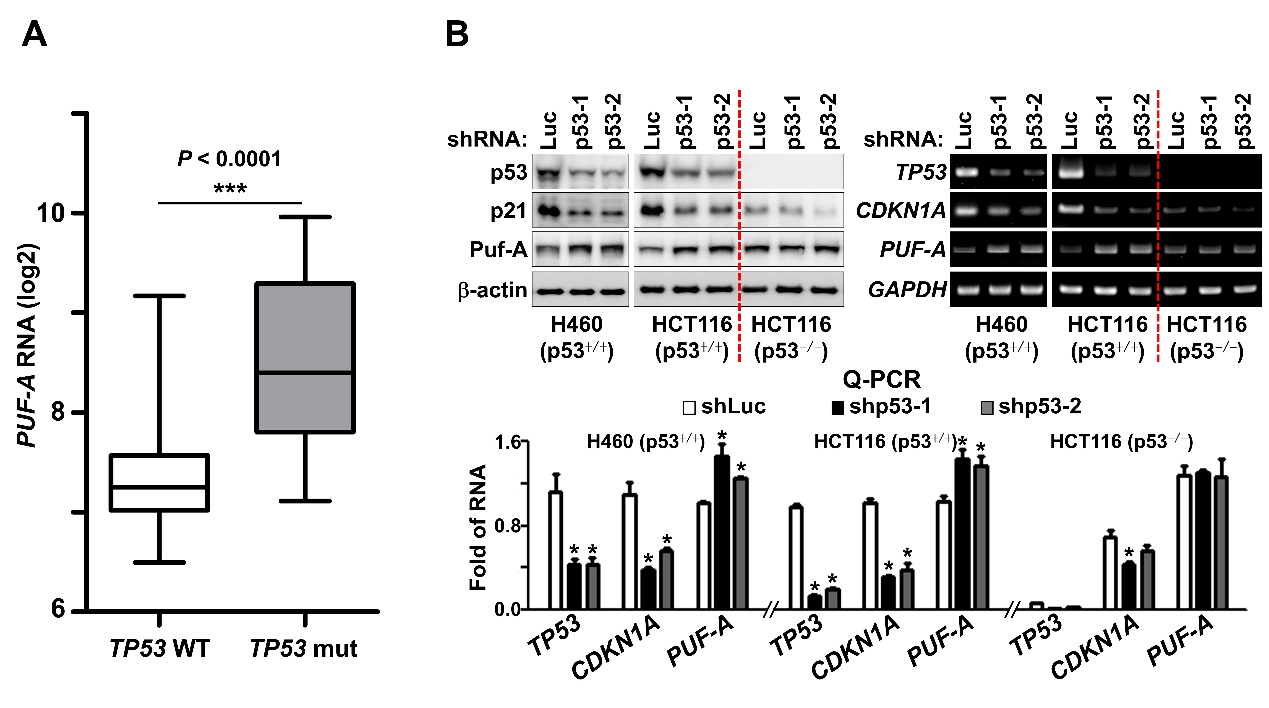


**Figure S6. Loss of p53 function induces Puf-A expression**

Western blot, RT-PCR, and Q-PCR analyses were performed for the expressions of Puf-A (*PUF-A*), p53 (*TP53*), and p21 (*CDKN1A*) in p53^+/+^-H460 and -HCT116 cells after transduction with control shLuc, shp53-1, and shp53-2 viruses. β-actin and GAPDH were used as internal controls. In Q-PCR, mean ± SD values (n = 3) are shown. * *p* < 0.05 (one-way ANOVA).

**Figure S7**

**Figure S7. p53 hotspot mutants show little p53 transactivation.**

Three p53 hotspot mutants, R175H, R248W and R273H, were generated by site-direct mutagenesis and then cloned into HA-tag expression vector. These hotspot mutants along with pp53-TA-luc vector, containing p53 cis-acting element, were transfected in H1299 and HCT116 p53-/- cells, respectively. Normalized activity of luciferase reporter with a control plasmid was shown. Mean ± SD was presented

**Figure S8**

**Figure S8. p53 hotspot mutants could not repress Puf-A promoter activity.**

The pRE-1 construct drove by DNA fragment containing the p53 binding site p53RE-1 (-720 to -700 nt). The pRE-1m construct consisted of the region with mutations. The Puf-A promoter, pRE-1 and pRE-1m constructs along with three p53 hotspot mutants were transfected in H1299 and HCT116 p53-/- cells, respectively. Normalized activity of luciferase reporter with a control plasmid was shown. Mean ± SD was presented.

**Figure S9**


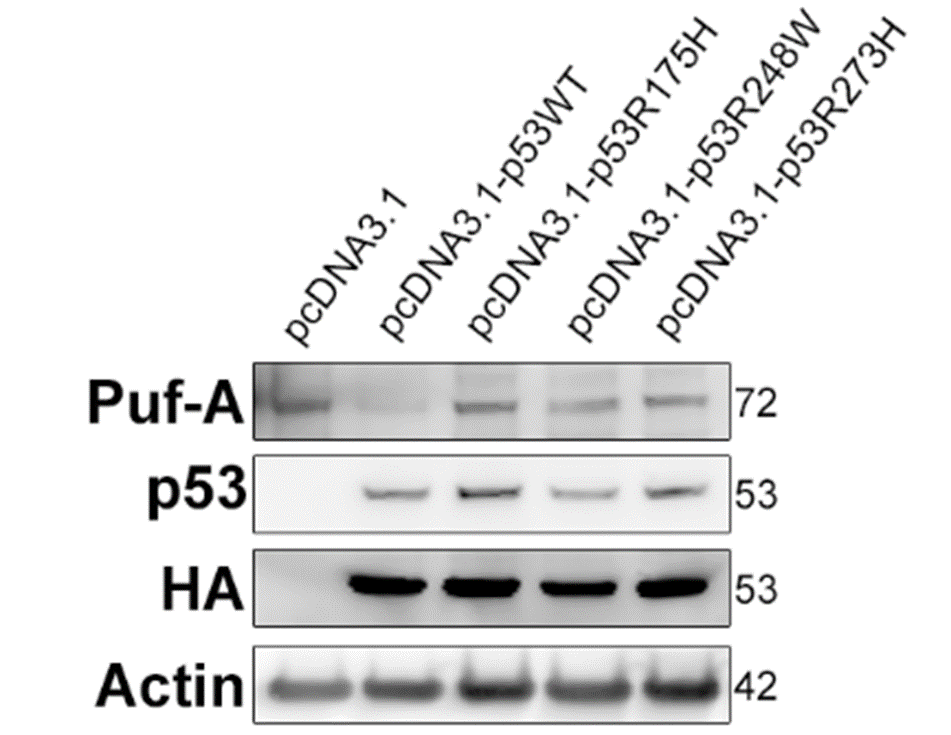


**Figure S9. p53 mutants have no repression effect on Puf-A expression.** H1299 cells (p53^null^) were transfected with p53 mutants, including p53R175H, p53R248W, and p53R273H or p53 wild-type. Western blot analysis of Puf-A, p53, HA, and actin was performed after transfected with the indicated p53 clones.

**Figure S10**


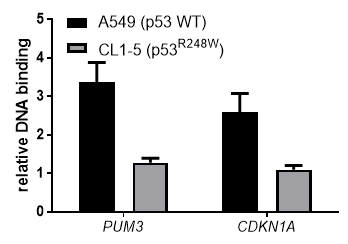


**Figure S10. ChIP-qPCR analysis for binding of p53 to the response element regions of either *PUM3* or *CDKN1A* in A549 and CL1-5 cells.** ChIP-qPCR was used to amplify chromatin derived from immunoprecipitations with anti-TP53 antibody. Each data point represents the average of three independent ChIP experiments.

**Figure S11
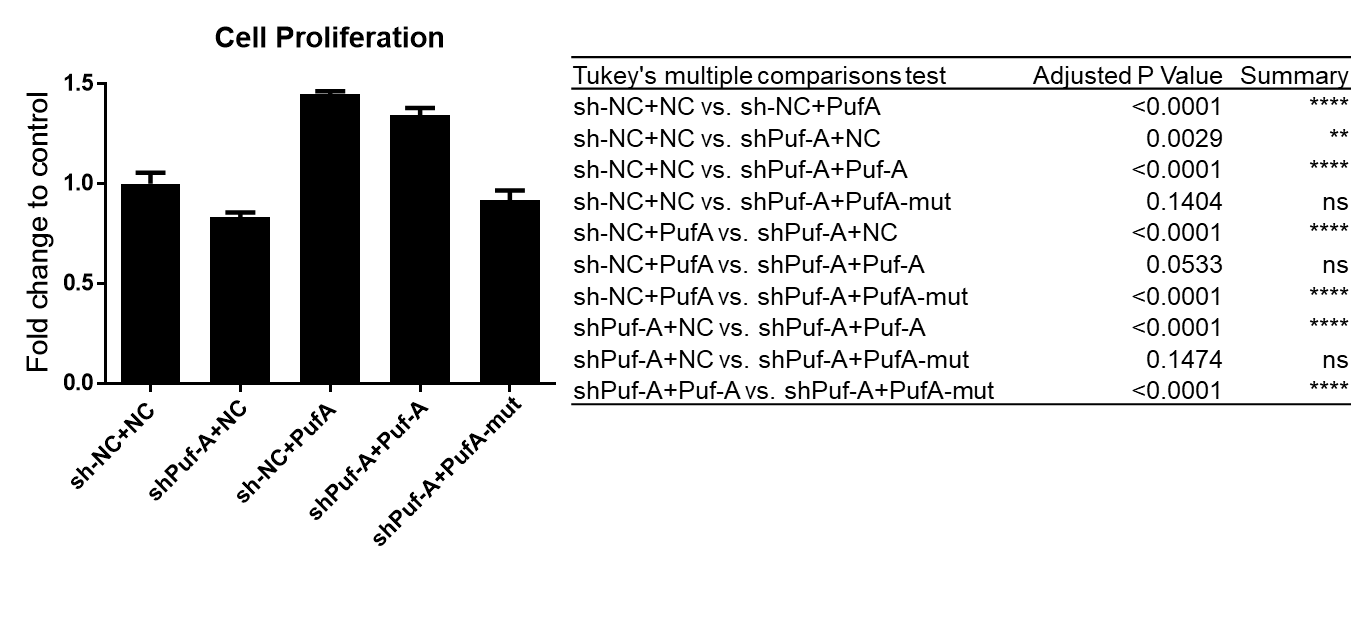
Figure S11. Expression of Puf-A rescued the phenotype of Puf-A knockdown H1299 cells.** H1299 cells were transfected with control (sh-NC) or shPuf-A plasmids. Forty eight hours later, these cells were transfected with 100 ng of plasmids encoding either negative control (NC), Puf-A, or Puf-A mutant deficient in binding to NPM1 (PufA460). After 72 hr incubation, cell proliferation was determined by an AlamarBlue assay. Bars, mean ± SD.

**Figure S12**

**Figure S12. Puf-A increased proliferation of A549 cells.**(A) A549 cells were seeding into a 96-well plate and then transfected with Puf-A and control plasmids with the indicated concentrations for 48hrs. (B) A549 cells were transfected with 100 ng Puf-A and control plasmids for 48 and 72 hrs. After transfection, cell proliferation was evaluated with AlamarBlue assay. * P < 0.05; ** P < 0.01; *** P < 0.001, compared with control.

**Figure S13**


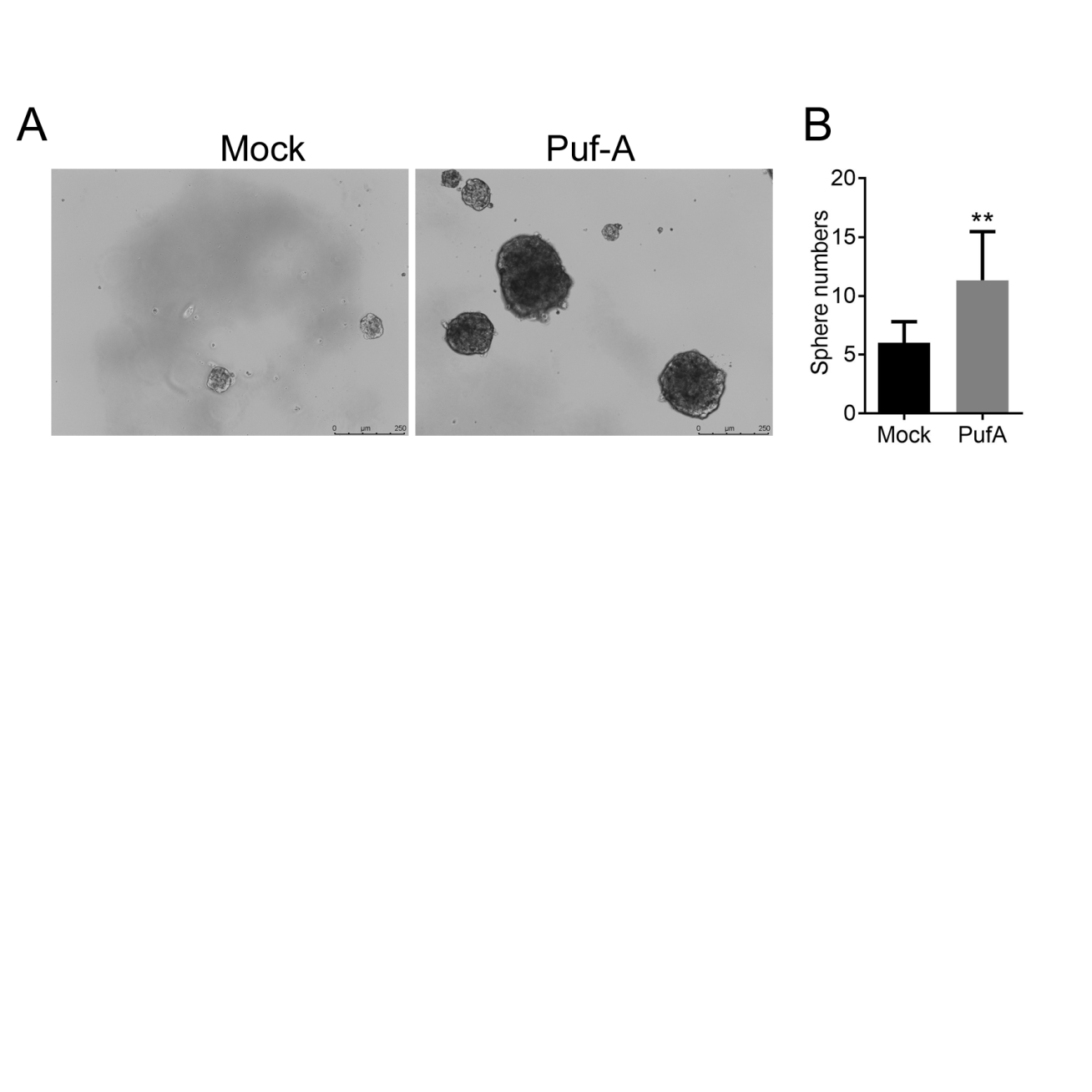


**Figure S13. Puf-A enhanced mammosphere formation.**

A549 cells were transfected with control and Puf-A-encoding plasmids and cultured for 2 weeks. (A) Phase-contrast images of mammospheres of A549 cells with/without Puf-A overexpression. Bar, 250 μm. (B) Mammosphere number in mock and Puf-A over-expressing A549 cells. Mean ± SDs, ** P < 0.001.

**Figure S14**


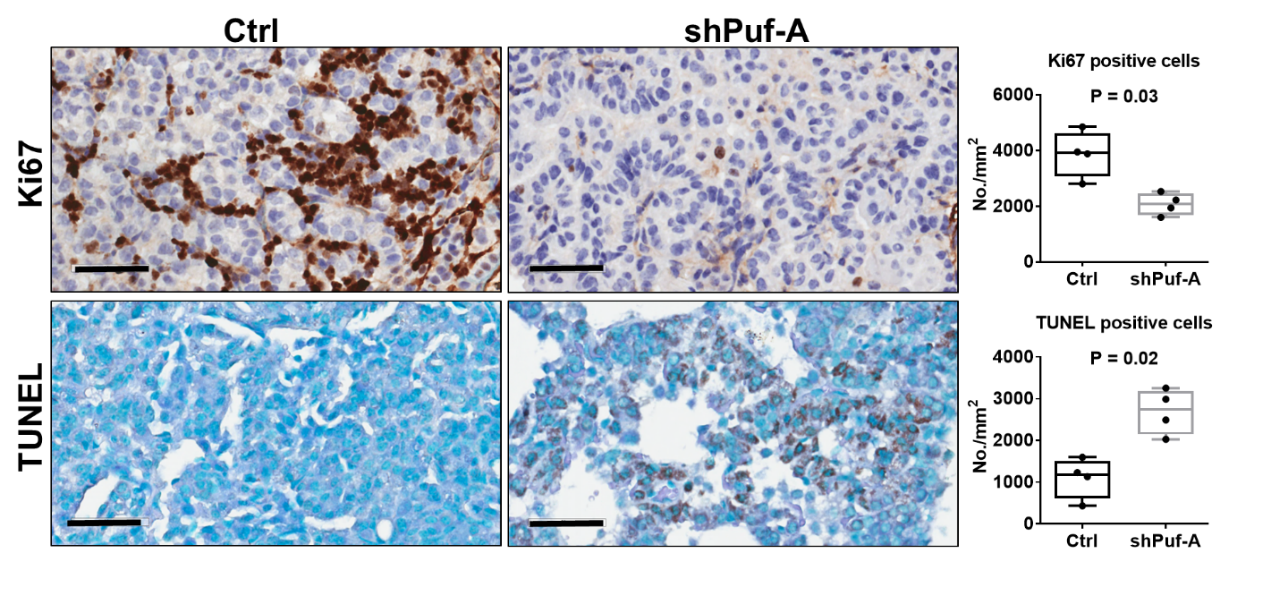


**Figure S14. Puf-A silencing increases apoptosis and decreases proliferation of tumor cells in CCSP-rtTA/TetO-Cre/KRAS^G12D^/p53^Flox/Flox^ mice.** Lentivirus expressing Puf-A shRNA or control shLacZ was delivered intranasally (20 μL, 2×10^7^ infection units) to the mice (n = 4 each) twice a week for two weeks. At week six, mice were sacrificed and lung was excised for IHC and TUNEL assays. Upper panel: Ki67 immunohistochemical staining; lower panel: TUNEL assay. Images of the IHC and TUNEL assays were acquired, and randomly selected areas from each tumor were analyzed. The number of Ki67 or TUNEL positive cells was counting using StrataQuest. The student t-test was used for comparison. Scale bars: 50 μm (original magnification 40X).

**Figure S15**


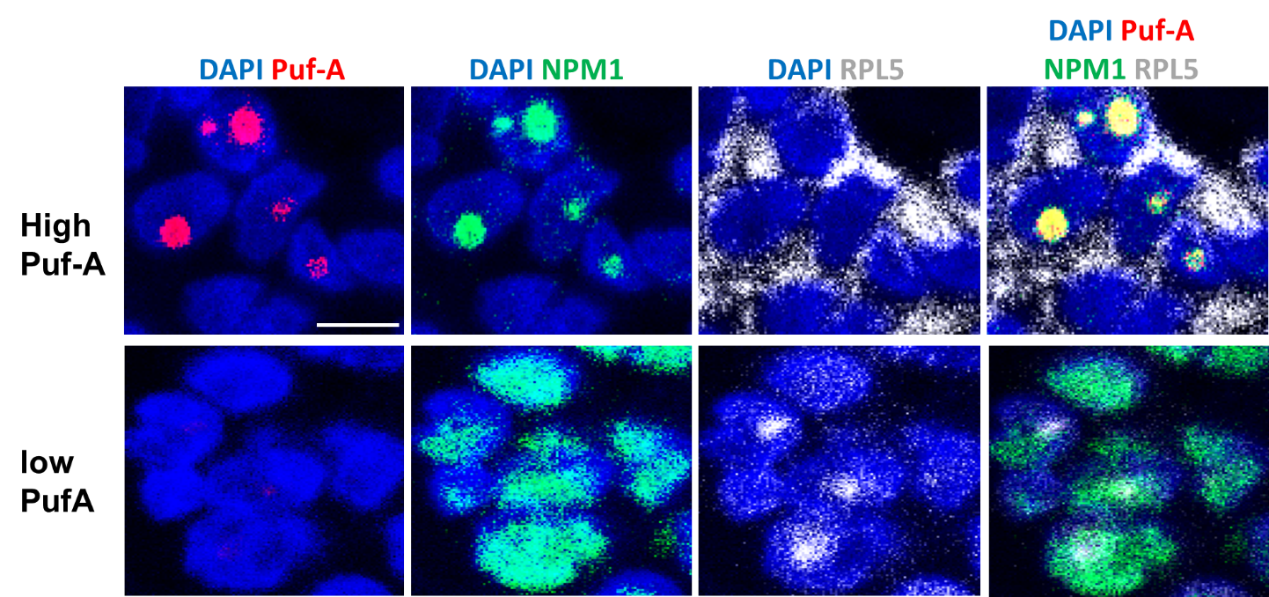


**Figure S15. The impact of Puf-A on cellular localization of NPM1 and ribosomal protein L5 (RPL5) in NSCLC tumor specimen.** Representative photomicrographs of Opal multiplex immunofluorescence assay for Puf-A (red), NPM1 (green), RPL5 (white), and DAPI on NSCLC samples with high (n = 3) or low (n = 3) Puf-A expression. Scale bars: 20 μm.

**Figure S16, related to Figure 6**


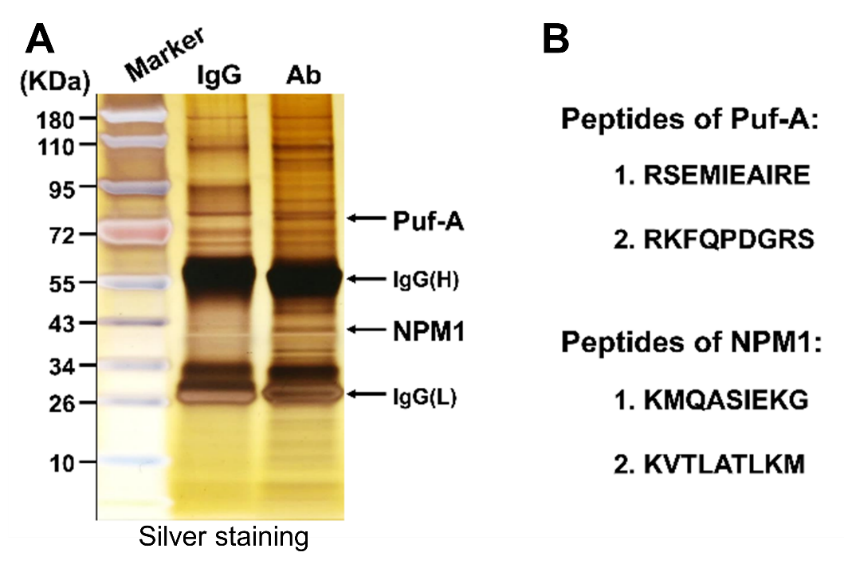


**Figure S16. Immunoprecipitation and amino acid analyses of Puf-A interacting protein**

(A) Cell extracts from H1299 cells were immunoprecipitated by anti-Puf-A antibody. IgG was used as a negative control. Silver staining was carried out to show potential Puf-A associated proteins. (B) Amino acid sequences of the tryptic peptides were detected in mass spectrometric analysis of the immunoprecipitation complex obtained from silver stained gel. These results confirm the identities of the Puf-A and NPM1 fragments, respectively.

**Figure S17**

**A**


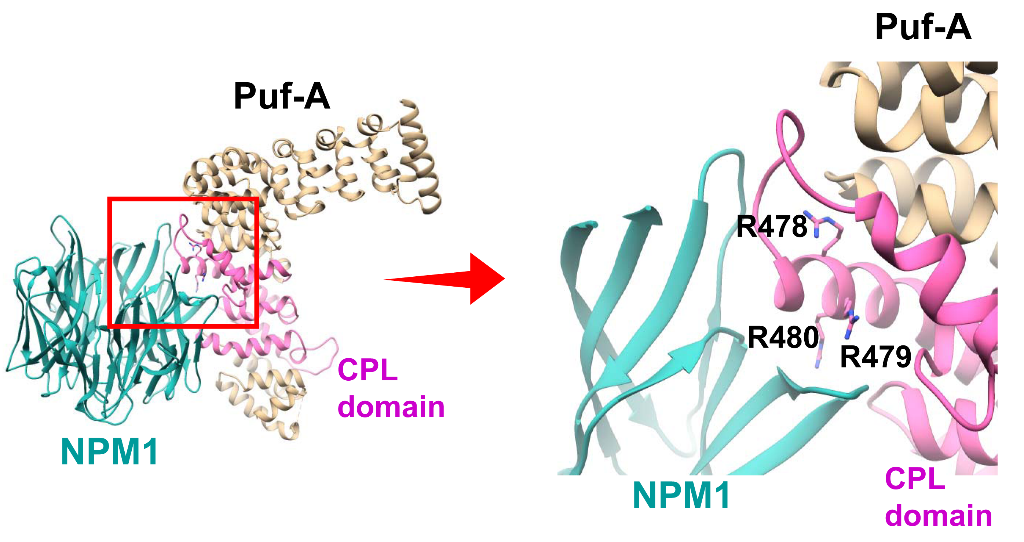


**B**


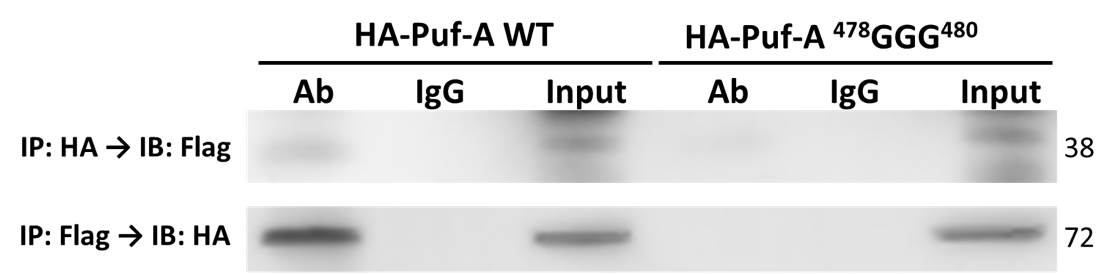


**Figure S17. Interaction of Puf-A mutant and NPM1**

(A) Computer modeling of the binding region of Puf-A to NPM1. (B) H1299 cells were co-transfected with flag-tagged NPM1 (flag-NPM1) and either HA-tagged Puf-A (HA-Puf-A) or HA-tagged Puf-A mutant (^478^GGG^480^). Cell lysates of the transfected cells was co-immunoprecipitated with antibody against HA and then immunoblotted with anti-flag and vice versa.

**Figure S18**


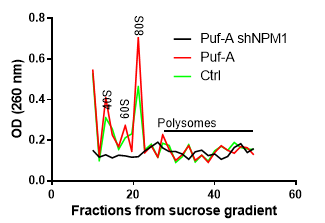


**Figure S18. Interaction between Puf-A and NPM1 is crucial for ribosome biogenesis.** Puf-A was overexpressed in HCT116 (p53 WT) cells with or without silencing NPM1 using shNPM1. Sucrose gradient centrifugation was performed and ribosomal subunits in the fractionated gradients were measure by OD 260 nm.

**II. Supplementary Tables**

**Table S1. Clinical characteristics of the patients with lung cancer**

| Sample No | SEX | Histology | pStage | pTNM | Grade | Puf-A IHC (H-SCORE) | p53 IHC |
| --- | --- | --- | --- | --- | --- | --- | --- |
| 1 | F | ADENOCARCINOMA | 4 | pT2aN1M1b | 2 | 150 | High |
| 2 | F | ADENOCARCINOMA | 12 | pT2aN0M0 | 2 | 190 | negative |
| 3 | M | ADENOCARCINOMA | 11 | pT1N0Mx | 3 | 120 | negative |
| 4 | M | ADENOCARCINOMA | 31 | pT2bN2M0 | 3 | 75 | Low |
| 5 | F | ADENOCARCINOMA | 31 | pT2aN2M0 | 2 | 140 | High |
| 6 | F | ADENOCARCINOMA | 31 | pT2aN2M0 | 2 | 40 | Low |
| 7 | M | ADENOCARCINOMA | 11 | pT1bN0M0 | 1 | 100 | Low |
| 8 | M | ADENOCARCINOMA | 12 | pT2aN0M0 | 1 | 60 | Negative |
| 9 | F | ADENOCARCINOMA | 11 | pT1N0Mx | 2 | 60 | High |
| 10 | F | ADENOCARCINOMA | 31 | pT1N2Mx | 2 | 40 | High |
| 11 | F | ADENOCARCINOMA | 11 | pT1bN0M0 | 2 | 180 | Low |
| 12 | F | ADENOCARCINOMA | 11 | ypT1aN0M0 | 2 | 140 | Negative |
| 13 | M | ADENOCARCINOMA | 11 | pT1N0MX | 3 | 120 | Negative |
| 14 | F | ADENOCARCINOMA | 11 | pT1bN0M0 | 2 | 240 | High |
| 15 | M | ADENOCARCINOMA | 22 | pT2bN1M0 | 3 | 100 | High |
| 16 | F | ADENOCARCINOMA | 21 | pT2aN1M0 | 2 | 0 | Low |
| 17 | M | ADENOCARCINOMA | 12 | pT2N0Mx | 2 | 40 | High |
| 18 | F | ADENOCARCINOMA | 22 | pT2N1Mx | 1 | 5 | High |
| 19 | F | ADENOCARCINOMA | 11 | pT1bN0M0 | 1 | 10 | Low |
| 20 | F | ADENOCARCINOMA | 12 | pT2N0Mx | 3 | 160 | Negative |
| 21 | F | ADENOCARCINOMA | 31 | pT2N2Mx | 3 | 100 | High |
| 22 | M | ADENOCARCINOMA | 11 | pT1N0Mx | 1 | 40 | Negative |
| 23 | F | ADENOCARCINOMA | 31 | pT3N2M0 | 1 | 0 | Low |
| 24 | M | ADENOCARCINOMA | 31 | pT2N2M0 | 2 | 140 | Low |
| 25 | M | ADENOCARCINOMA | 12 | pT2aN0M0 | 2 | 80 | Low |
| 26 | M | ADENOCARCINOMA | 31 | pT1bN2M0 | 1 | 80 | High |
| 27 | F | ADENOCARCINOMA | 32 | pT4N2M0 | 1 | 20 | Negative |
| 28 | F | ADENOCARCINOMA | 11 | ypT1aN0M0 | 1 | 0 | Low |
| 29 | F | ADENOCARCINOMA | 21 | pT1bN1M0 | 1 | 20 | Negative |
| 30 | M | ADENOCARCINOMA | 31 | pT2aN2M0 | 2 | 70 | Negative |
| 31 | F | ADENOCARCINOMA | 11 | pT1bN0M0 | 1 | 40 | Negative |
| 32 | M | ADENOCARCINOMA | 12 | pT2aN0M0 | 2 | 40 | High |
| 33 | F | ADENOCARCINOMA | 4 | pT2N0M1 | 2 | 70 | Low |
| 34 | F | ADENOCARCINOMA | 12 | pT2N0MX | 1 | 90 | Low |
| 35 | F | ADENOCARCINOMA | 32 | ypT4N1M0 | 1 | 0 | Low |
| 36 | M | ADENOCARCINOMA | 12 | ypT2aN0MX | 2 | 60 | Low |
| 37 | F | ADENOCARCINOMA | 12 | pT2aN0M0 | 2 | 30 | High |
| 38 | M | ADENOCARCINOMA | 12 | pT2aN0M0 | 2 | 30 | Low |
| 39 | M | ADENOCARCINOMA | 11 | pT1aN0M0 | 1 | 40 | Low |
| 40 | M | ADENOCARCINOMA | 11 | pT1bN0M0 | 1 | 20 | Low |
| 41 | M | ADENOCARCINOMA | 31 | pT2aN2M0 | 1 | 220 | High |
| 42 | F | ADENOCARCINOMA | 22 | pT2bN1M0 | 2 | 45 | Low |
| 43 | M | ADENOCARCINOMA | 31 | ypT2aN2M0 | 2 | 65 | Low |
| 44 | M | ADENOCARCINOMA | 31 | pT3N2M0 | 2 | 100 | Low |
| 45 | F | ADENOCARCINOMA | 11 | pT1bN0M0 | 1 | 10 | High |
| 46 | F | ADENOCARCINOMA | 12 | pT2aN0MX | 1 | 250 | High |
| 47 | F | ADENOCARCINOMA | 31 | pT2bN2M0 | 1 | 110 | Negative |
| 48 | F | ADENOCARCINOMA | 12 | ypT2aN0M0 | 2 | 210 | High |
| 49 | F | ADENOCARCINOMA | 31 | pT1bN2M0 | 1 | 20 | Low |
| 50 | M | ADENOCARCINOMA | 31 | pT3N1M0 | 2 | 140 | High |
| 51 | M | ADENOCARCINOMA | 11 | pT1bN0M0 | 1 | 20 | Low |
| 52 | F | ADENOCARCINOMA | 12 | pT2aN0M0 | 1 | 60 | Low |
| 53 | F | ADENOCARCINOMA | 11 | pT1aN0M0 | 1 | 60 | Low |
| 54 | F | ADENOCARCINOMA | 12 | pT2aN0M0 | 1 | 90 | Low |
| 55 | F | ADENOCARCINOMA | 11 | pT1bN0M0 | 1 | 130 | High |
| 56 | F | ADENOCARCINOMA | 11 | pT1aN0M0 | 1 | 40 | Low |
| 57 | F | ADENOCARCINOMA | 11 | pT1bN0M0 | 2 | 130 | Low |
| 58 | F | ADENOCARCINOMA | 11 | pT1bN0 | 1 | 20 | Low |
| 59 | F | ADENOCARCINOMA | 21 | pT2aN1 | 2 | 75 | Low |
| 60 | F | ADENOCARCINOMA | 11 | pT1bN0 | 1 | 20 | Low |
| 61 | F | ADENOCARCINOMA | 31 | pT2aN2 | 2 | 150 | High |
| 62 | M | ADENOCARCINOMA | 21 | pT2aN1M0 | 3 | 160 | High |
| 63 | F | ADENOCARCINOMA | 31 | pT1bN2M0 | 2 | 230 | High |
| 64 | F | ADENOCARCINOMA | 31 | pT2aN2M0 | 2 | 130 | High |
| 65 | F | LARGE CELL CARCINOMA | 21 | pT2bN0M0 | 4 | 110 | High |
| 66 | M | LARGE CELL CARCINOMA | 12 | pT2aN0M0 | 4 | 120 | High |
| 67 | M | LARGE CELL CARCINOMA | 12 | pT2aN0MX | 4 | 110 | Low |
| 68 | M | SARCOMATOID CARCINOMA | 12 | pT2aN0M0 | 3 | 110 | Negative |
| 69 | F | SQUAMOUS CELL CARCINOMA | 11 | pT1bN0MX | 3 | 130 | High |
| 70 | M | SQUAMOUS CELL CARCINOMA | 12 | pT2aN0M0 | 2 | 50 | Negative |
| 71 | M | SQUAMOUS CELL CARCINOMA | 11 | pT1N0M0 | 3 | 160 | High |
| 72 | F | SQUAMOUS CELL CARCINOMA | 31 | pT2aN2M0 | 4 | 80 | Low |
| 73 | M | SQUAMOUS CELL CARCINOMA | 31 | pT2bN2M0 | 3 | 150 | High |
| 74 | M | SQUAMOUS CELL CARCINOMA | 21 | pT2bN0M0 | 2 | 10 | Negative |
| 75 | M | SQUAMOUS CELL CARCINOMA | 11 | pT1bN0M0 | 2 | 60 | High |
| 76 | M | SQUAMOUS CELL CARCINOMA | 22 | pT2bN1M0 | 2 | 170 | Low |
| 77 | M | SQUAMOUS CELL CARCINOMA | 11 | pT1bN0M0 | 3 | 220 | Negative |
| 78 | M | SQUAMOUS CELL CARCINOMA | 12 | pT2aN0M0 | 2 | 260 | Negative |
| 79 | M | SQUAMOUS CELL CARCINOMA | 31 | pT2aN2 | 2 | 230 | Negative |
| 80 | M | SQUAMOUS CELL CARCINOMA | 11 | pT2aN0M0 | 2 | 180 | High |
| 81 | F | SQUAMOUS CELL CARCINOMA | 31 | pT2N2Mx | 2 | 100 | High |
| 82 | M | SQUAMOUS CELL CARCINOMA | 21 | pT2N1M0 | 3 | 230 | High |

**Table S2. Overall survival of the patients with stage I lung cancer**

| No | pStage | pTNM | Puf-A_HSCORE | Survival | OS | Puf-A_hscore_HL2 |
| --- | --- | --- | --- | --- | --- | --- |
| 1 | stage 1 | N0 | 190 | death | 2616 | stage 1 Puf-A High |
| 2 | stage 1 | N0 | 120 | death | 1694 | stage 1 Puf-A High |
| 3 | stage 1 | N0 | 100 | death | 1905 | stage 1 Puf-A High |
| 4 | stage 1 | N0 | 60 | death | 1001 | stage 1 Puf-A High |
| 5 | stage 1 | N0 | 60 | death | 1162 | stage 1 Puf-A High |
| 6 | stage 1 | N0 | 180 | alive | 2626 | stage 1 Puf-A High |
| 7 | stage 1 | N0 | 140 | death | 458 | stage 1 Puf-A High |
| 8 | stage 1 | N0 | 120 | alive | 549 | stage 1 Puf-A High |
| 9 | stage 1 | N0 | 180 | death | 394 | stage 1 Puf-A High |
| 10 | stage 1 | N0 | 240 | death | 1021 | stage 1 Puf-A High |
| 13 | stage 1 | N0 | 160 | alive | 1870 | stage 1 Puf-A High |
| 16 | stage 1 | N1 | 160 | alive | 3090 | stage 1 Puf-A High |
| 17 | stage 1 | N0 | 80 | alive | 2094 | stage 1 Puf-A High |
| 20 | stage 1 | N0 | 110 | death | 168 | stage 1 Puf-A High |
| 22 | stage 1 | N4 | 90 | death | 1461 | stage 1 Puf-A High |
| 23 | stage 1 | N0 | 60 | death | 1998 | stage 1 Puf-A High |
| 24 | stage 1 | N2 | 120 | death | 683 | stage 1 Puf-A High |
| 29 | stage 1 | N4 | 60 | death | 856 | stage 1 Puf-A High |
| 31 | stage 1 | N0 | 250 | alive | 1660 | stage 1 Puf-A High |
| 32 | stage 1 | N0 | 110 | alive | 1572 | stage 1 Puf-A High |
| 33 | stage 1 | N0 | 130 | alive | 1514 | stage 1 Puf-A High |
| 34 | stage 1 | N1 | 210 | alive | 1519 | stage 1 Puf-A High |
| 36 | stage 1 | N0 | 60 | alive | 1360 | stage 1 Puf-A High |
| 37 | stage 1 | N0 | 60 | alive | 1308 | stage 1 Puf-A High |
| 38 | stage 1 | N0 | 90 | alive | 1135 | stage 1 Puf-A High |
| 39 | stage 1 | N0 | 130 | alive | 1243 | stage 1 Puf-A High |
| 41 | stage 1 | N0 | 130 | alive | 1158 | stage 1 Puf-A High |
| 44 | stage 1 | N0 | 220 | alive | 1064 | stage 1 Puf-A High |
| 45 | stage 1 | N0 | 260 | alive | 870 | stage 1 Puf-A High |
| 11 | stage 1 | N0 | 40 | alive | 3206 | stage 1 Puf-A low |
| 12 | stage 1 | N0 | 10 | NA | NA | stage 1 Puf-A low |
| 14 | stage 1 | N0 | 40 | NA | NA | stage 1 Puf-A low |
| 15 | stage 1 | N0 | 50 | death | 198 | stage 1 Puf-A low |
| 18 | stage 1 | N0 | 0 | alive | 2937 | stage 1 Puf-A low |
| 19 | stage 1 | N0 | 40 | alive | 2575 | stage 1 Puf-A low |
| 21 | stage 1 | N1 | 40 | death | 586 | stage 1 Puf-A low |
| 25 | stage 1 | N3 | 30 | alive | 2577 | stage 1 Puf-A low |
| 26 | stage 1 | N0 | 30 | alive | 2520 | stage 1 Puf-A low |
| 27 | stage 1 | N0 | 40 | alive | 2245 | stage 1 Puf-A low |
| 28 | stage 1 | N0 | 20 | alive | 2238 | stage 1 Puf-A low |
| 30 | stage 1 | N0 | 10 | alive | 1995 | stage 1 Puf-A low |
| 35 | stage 1 | N0 | 20 | alive | 1480 | stage 1 Puf-A low |
| 40 | stage 1 | N0 | 40 | alive | 1198 | stage 1 Puf-A low |
| 42 | stage 1 | N0 | 20 | alive | 1151 | stage 1 Puf-A low |
| 43 | stage 1 | N0 | 20 | alive | 1023 | stage 1 Puf-A low |

**III. Supplementary materials and methods**

**Plasmid construction, lentiviral production, and cell transduction**

The lentiviral shRNAs used for gene silencing were obtained from the RNAi core facility of Academia Sinica, Taiwan. Full-length cDNA of human *TP53* (from A549 cells) was cloned into a pSin-EF2-IRES-Pur (Addgene, MA, USA) lentiviral or pLenti6/V5-TOPO (Invitrogen, CA, USA) lentiviral vector. The primers for plasmid construction and oligo sequences and gene IDs of shRNAs are listed in the supplementary materials and methods. Lentivirus was produced by transient co-transfection of pCMVΔ8.91 (10 µg), pMD.G-VSV-G (10 µg), and the lentiviral expression vector (10 µg) into 293T cells. Viral supernatants were concentrated by ultracentrifugation to produce viruses with titers of 1×10^8^ infection units/mL. Various cells were infected with lentivirus in the presence of 8 μg/mL polybrene (Millipore, MA, USA).

**qPCR**

Total RNA was extracted using an RNeasy spin column (Qiagen, MD, USA). One microgram of total RNA was used for cDNA synthesis using a Sensiscript Reverse Transcriptase kit (Qiagen) according to the manufacturer’s suggestions. A real-time quantitative PCR system was used to quantify the relative mRNA levels in the samples as described [[1](#_ENREF_1)]. The primers were designed for a Fast SYBR Green system (Applied Biosystems, CA, USA). Briefly, the PCR was performed in a thermal cycler (ABI PRISM 7900 Sequence Detection System; Applied Biosystems) with the following sequence: reaction at 50°C for 2 min and at 95°C for 10 min, and subsequently, the PCR was repeated for 40 cycles of denaturation at 95°C for 15 s and annealing and extension at 60°C for 1 min. The authenticity of PCR products was verified with 1.5% agarose gel electrophoresis. The relative concentration of each mRNA was calculated using the ΔΔ*C*_T_ method according to the manufacturer’s user manual. The Δ*C*_T_ value of the study sample was calculated using the following formula: Δ*C*_T_ = *C*_T_ of target gene−*C*_T_ of GAPDH (designated as ‘sample Δ*C*_T_’). Similarly, for the Δ*C*_T_ value of the calibrator, *C*_T_ values of the target gene and GAPDH were obtained, and the Δ*C*_T_ was calculated (designated as ‘calibrator Δ*C*_T_’). Finally, the ΔΔ*C*_T_ value was calculated using the following formula: ΔΔ*C*_T_ = Δ*C*_T_ (sample) −Δ*C*_T_ (calibrator); the relative value of each mRNA was calculated using the formula: 2^−ΔΔ^*^C^*^T^.

**Western blot analysis**

For the preparation of whole-cell lysates, cells were lysed in modified RIPA buffer (Millipore, USA) and 1X protease inhibitor cocktail (Roche, Germany). Protein concentrations were measured using Bio-Rad protein assay dye reagent (Bio-Rad, CA, USA). Proteins (40 μg) were separated on 10% SDS-PAGE gels and transferred onto PVDF membranes (Millipore, USA). The membranes were probed with antibodies recognizing Puf-A (monoclonal antibody generated by our lab), p53 (sc-126, Santa Cruz), p21 (sc-397, Santa Cruz), caspase-3 (IMG-144A, IMGENEX, CA, USA), PARP (#9542S, Cell Signaling Technology, MA, USA), HA (sc-7392, Santa Cruz), flag (F3165, Sigma), NPM1 (sc-5564, Santa Cruz), S6 (sc-74459, Santa Cruz, USA), and L5 (ab157099, Abcam), and β-actin (A2228, Sigma) and then incubated with the appropriate horseradish peroxidase-conjugated secondary antibodies. Bands were visualized using enhanced chemiluminescence reagents (PerkinElmer, USA).

**Cell cycle analysis**

After transduction, cells were resuspended in PBS and fixed with ethanol overnight at −20°C. Cells were then resuspended in PBS and treated with 100 µg/mL ribonuclease A (bovine pancreas; Sigma), 0.1% Triton-X100, and 40 µg/mL propidium iodide for 30 min at 37°C. Cell cycle distribution was detected with a FACSCalibur flow cytometer and analyzed by the ModFit LT program (Verity Software House, Topsham, ME, USA).

**Cell proliferation**

Cell proliferation was determined using a real-time monitoring assay based on variation of electric impedance (xCELLigence Technology, Roche). Briefly, after transduction, cells were seeded in an E-plate 96 plugged into an RTCA (Real-Time Cell Analysis) monitor within a CO_2_ incubator. Cell anchorage induces variations in electrical impedance that allows monitoring cell proliferation, because increases in electric impedance are correlated to proportional increases in cell proliferation. After normalization of electric impedance 24 h post-seeding, the cell proliferation index reflecting the slope of the growth curves was calculated by RTCA software (Roche).

**Co-immunoprecipitation**

The whole-cell lysates from various cells after transfection or transduction were lysed in modified RIPA buffer (Millipore) and then centrifuged at 12,000 rpm for 15 min at 4°C. The lysates were subjected to immunoprecipitation with an anti-HA (sc-7392, Santa Cruz), anti-flag (F3165, Sigma), anti-Puf-A (monoclonal antibody), anti-NPM1 (sc-5564, Santa Cruz) antibodies and normal mouse or rabbit IgG (sc-2025 and sc-2027, Santa Cruz) for immunoprecipitation. Then, these immunocomplexes were analyzed as described in the western blot assay.

**Analysis of publicly available datasets for *PUF-A* RNA expression**

To analyze the expression of *PUF-A* RNA in human lung cancer, we used GSE83227 and GSE68571 datasets [[2](#_ENREF_2), [3](#_ENREF_3)].

**Genotyping**

Amplification of PCR products for CCSP-rtTA and TetO-Cre transgenic mice and p53^flox/flox^ knock-in mice was performed as follows: denaturation at 95°C for 5 min; 30 cycles of denaturation at 94°C for 30 s, annealing at 60°C for 30 s, and extension at 72°C for 30 s; followed by a 5-min extension at 72°C. For LSL-Kras^G12D^ knock-in mice, amplification was performed as follows: denaturation at 95°C for 5 min; 40 cycles of denaturation at 94°C for 30 s, annealing at 69°C for 45 s, and extension at 72°C for 45 s; followed by a 5-min extension at 72°C.

**Intranasal delivery of lentivirus expressing Puf-A shRNA**

Sample size was chosen based on our experience. Two weeks after administration of doxycycline, CCSP-rtTA/TetO-Cre/LSL-Kras^G12D^/p53^flox/flox^ mice were anesthetized with isoflurane (Halocarbon, GA, USA). We pooled the mice together, and then assigned them blindly for treatment and control groups. The lentivirus expressing Puf-A shRNAs or control shLacZ (20 μL, 1×10^9^ infection units/mL) was delivered intranasally to mice twice a week for two weeks. Six weeks after Kras^G12D^ activation and p53 deletion, mice were sacrificed and the tumor foci in the lungs were examined. H&E-stained sections of lungs were imaged on Aperio ScanScope AT Turbo scanner (Leica Biosystems, Germany), and the tumor number and size (diameter) were measured by the Snapshot software. Percentages of adenomas and ADCs in the lung tissue sections were determined using Metamorph^TM^ software (Molecular Devices, CA, USA).

***PUF-A* promoter activity**

To investigate whether p53 regulates *PUF-A* promoter activity, a 12130 bp DNA fragment of the *PUF-A* locus from -6508 to +5622 nt was cloned into a pGL3-basic vector (Promega, WI, USA). Cells were seeded in a 6-well plate and co-transfected with p53-expressing vector and pGL3-*PUF-A*-Luc for 72 h using Lipofectamine 2000 (Invitrogen). The *PUF-A* promoter activities were measured using a Dual-Luciferase® Reporter Assay System (Promega). The transfection efficiency was normalized by co-transfection with pRL-TK as an internal control.

**Sucrose gradient fractionation and polysome profile assay**

The sucrose gradient fractionation of ribosomal fractions was performed as described [[4](#_ENREF_4)]. After transduction, cells were lysed on ice in 200 μL of lysis buffer (20 mM Tris pH 7.5, 200 mM NaCl, 15 mM MgCl_2_, 1 mM DTT, 8% glycerol, 1% Triton X-100, 100 μg/mL cycloheximide, 100 μg/mL heparin, and 200 units/mL SUPERaseIn (Ambion, life technologies, CA, USA). After lysis, nuclei and membrane debris were removed by centrifugation (1,300 g for 5 min at 4°C, and then 14,000 rpm for 5 min at 4°C). The supernatant was layered onto a linear sucrose gradient (7–47% sucrose (w/v), 20 mM Tris pH 7.5, 100 mM NaCl, 15 mM MgCl_2_, 100 μg/mL cycloheximide) and centrifuged in an SW28 rotor for 4 h at 28,000 rpm at 4°C. Various fractions of ribosome extracts were examined with ultraviolet light at 260 nm. Equal volumes of protein extracts in various ribosomal fractions were precipitated with trichloroacetic acid and then analyzed with a western blot.

**Opal multiplex immunofluorescence staining**

| Staining sequence | Antibody | Clone (host) /Company | Dilution | Incubation | TSA dyes |
| --- | --- | --- | --- | --- | --- |
| **1** | Puf-A | homemade | 1:100 | 30 min | 570 |
| **2** | RPL5 | ab157099/ Abcam | 1:100 | 20 min | 520 |
| **3** | NPM1 | 3542S/ Cell signaling | 1:100 | 20 min | 690 |
| **4** | DAPI | Perkin Elmer Opal 7-color kit | 2 drops/ml | 5 min |  |

NSCLC tumor specimens were assessed with the Opal multiplex immunofluorescence staining, which provided simultaneous detection of Puf-A, NPM1, and RPL5. Slides were heated at 60°C for 30 minutes then Dewax (Leica Biosystems, Buffalo Grove, IL) was applied to remove paraffin. Antigen retrieval was performed using ER2 (Leica Biosystems) at 100°C for 30 minutes. Slides were washed and blocked with 3% H_2_O_2_ solution. The slide was incubated with Puf-A antibody followed by Opal polymer HRP Ms + Rb (Akoya Biosystems, Marlborough, MA). Signal was observed by incubating with tyramide signal amplification dye (Opal 7 color kit, Akoya Biosystems). After heating to strip the antibodies, the slides were blocked again using blocking solution. Repeat the above procedures to stain RPL5 and NPM1. Finally, counterstain with DAPI, and then a coverslip was mounted using Vectashield (Vector Laboratories, Burlingame, CA). Digital images of multiplex immunofluorescence staining were captured on a Leica TCS SP8 SMD confocal microscope using the Leica Imaging Suite software. Exposure time was manually adjusted for each fluorochrome to minimize autofluorescence. Image analysis was performed with Metamorph software (Molecular Devices, Inc.).

**TUNEL assay**

Lung tissues of lentivirus-treated mice were dissected, fixed in formalin, and subjected to paraffin embedding and sectioning. Paraffin sections were subjected to in situ apoptosis analysis following the manufacturer’s instruction (ab206386, Abcam). Images were captured on a Leica DM6000 microscope.

**Primer sequences for mouse genotyping**

| **Mice** | **genotyping sequences** |
| --- | --- |
| CCSP-rtTA transgenic mice | F: ACTGCCCATTGCCCAAACAC  R: AAAATCTTGCCAGCTTTCCCC |
| TetO-Cre transgenic mice | F: GCGGTCTGGCAGTAAAAACTATC  R: GTGAAACAGCATTGCTGTCACTT |
| LSL-Kras^G12D^ knock-in mice | F: AGCTAGCCACAATGGCTTGAGTAAGTCTGCA  R: GTCGACAAGCTCATGCGGGTG |
| p53^flox/flox^ knock-in mice | F: AAGGGGTATGAGGGACAAGG  R: GAAGACAGAAAAGGGGAGGG |

**Primer sequences for Q-PCR and RT-PCR**

| **Gene** | **Q-PCR primer sequence** |
| --- | --- |
| Human *PUF-A* (Q-PCR) | F: TGTGTGTTGGTGTCTGACATTCTG  R: AGCTGGCGATGGCATTCA |
| Human *TP53* (Q-PCR) | F: CCCCTCCTGGCCCCTGTCATCTTC  R: GCGCCTCACAACCTCCGTCAT |
| Human *CDKN1A* (Q-PCR) | F: GAGGCCGGGATGAGTTGGGAGGAG  R: CAGCCGGCGTTTGGAGTGGTAGAA |
| Human *NPM1* (Q-PCR) | F: CAAGAATCCTTCAAGAAACAGGA  R: ACTTCCTCCACTGCCAGAGA |
| Human *GAPDH* (Q-PCR) | F: AATCCCATCACCATCTTCCA  R: TGGACTCCACGACGTACTCA |
| Mouse *PUF-A* (Q-PCR) | F: GCCAGATGGTGAAAGTGATG  R: GCTCGAACCACGATGTCATA |
| Mouse *Trp53* (Q-PCR) | F: GTCTACGTCCCGCCATAAAA  R: AGGCAGTGAAGGGACTAGCA |
| Human *PUF-A* (RT-PCR) | F: TGTGTGTTGGTGTCTGACATTCTG  R: TAGAATTTCTATTCCTTTGCTGGT |
| Human *GAPDH* (RT-PCR) | F: ACCACAGTCCATGCCATC  R: TCCACCACCCTGTTGCTGTA |

**Primer sequences for plasmid construction**

| **cDNA** | **Primer sequence** | **expression vector** |
| --- | --- | --- |
| Human p53 | F: CCGCTCGAGATGGAGGAGCCGCAGTCAGATCCT  R: TCCCCGCGGTCAGTCTGAGTCAGGCCCTTCTGT | pLenti6/  V5-TOPO |
| Human HA-Puf-A  (underline: HA tag) | F: CCGCTCGAGGGACTAGTCATGTACCCTTATGACGTG CCCGATTACGCTATGGAAGTTAAAGGGAAAAAGCAATTC  R: CGCGGATCCCTATGTGCTCAGTTTTTCAAGTAGAAT | pSin-EF2-  IRES-Pur |
| Human flag-NPM1  (underline: flag tag) | F: GACTAGTCATGGACTACAAGGACGACGATGACAAG ATGGAAGATTCGATGGACATGGACATGAGC  R: GGAATTCCTTAAAGAGACTTCCTCCACTGCCAGAG ATC | pSin-EF2-  IRES-Pur |

**shRNAs for gene silencing**

| **shRNA** | **Oligo sequence** |
| --- | --- |
| control shLacZ (Gene ID: TRCN0000072224) | CCGGCGCGATCGTAATCACCCGAGTCTCGAGACTCGGGTGATTACGATCGCGTTTTTG |
| Mouse shPuf-A-1 (Gene ID: TRCN0000173994) | CCGGGCGGACCTAAAGTCACATCTACTCGAGTAGATGTGACTTTAGGTCCGCTTTTTTG |
| Mouse shPuf-A-2 (Gene ID: TRCN0000175742) | CCGGGCGACTTGGTTGAATTAAGTACTCGAGTACTTAATTCAACCAAGTCGCTTTTTTG |
| control shNC (Gene ID: TRCN0000231715) | CCGGCGCTGAGTACTTCGAAATGTCCTCGAGGACATTTCGAAGTACTCAGCGTTTTTG |
| Human shPuf-A-1 (Gene ID: TRCN0000280605) | CCGGGCCTAGCATAGTAAATGACAACTCGAGTTGTCATTTACTATGCTAGGCTTTTTG |
| Human shPuf-A-2 (Gene ID: TRCN0000280670) | CCGGCGTGTGATCCAGTGTTACATTCTCGAGAATGTAACACTGGATCACACGTTTTTG |
| Human shp53-1 (Gene ID: TRCN0000003753) | CCGGCGGCGCACAGAGGAAGAGAATCTCGAGATTCTCTTCCTCTGTGCGCCGTTTTT |
| Human shp53-2 (Gene ID: TRCN0000010814) | CCGGGAGGGATGTTTGGGAGATGTACTCGAGTACATCTCCCAAACATCCCTCTTTTT |

**IV. Supplementary references**

1. Lin RJ, Lin YC, Chen J, Kuo HH, Chen YY, Diccianni MB, et al. microRNA signature and expression of Dicer and Drosha can predict prognosis and delineate risk groups in neuroblastoma. Cancer Res. 2010;70:7841-50.

2. Bhattacharjee A, Richards WG, Staunton J, Li C, Monti S, Vasa P, et al. Classification of human lung carcinomas by mRNA expression profiling reveals distinct adenocarcinoma subclasses. Proc Natl Acad Sci U S A. 2001;98:13790-5.

3. Beer DG, Kardia SL, Huang CC, Giordano TJ, Levin AM, Misek DE, et al. Gene-expression profiles predict survival of patients with lung adenocarcinoma. Nat Med. 2002;8:816-24.

4. Xue S, Tian S, Fujii K, Kladwang W, Das R, Barna M. RNA regulons in Hox 5' UTRs confer ribosome specificity to gene regulation. Nature. 2015;517:33-8.
